# Size-Selective CO_2_ Activation at Rhodium Cluster Anions

**DOI:** 10.1021/jacs.6c05767

**Published:** 2026-07-01

**Authors:** Christian T. Haakansson, David J. Vesty, Peter T. Rubli, Peter D. Watson, Ellen A. Jones, Jasmin P. Justen, André Fielicke, Joost M. Bakker, Stuart R. Mackenzie

**Affiliations:** † Department of Chemistry, Chemistry Research Laboratory, 6396 University of Oxford, Mansfield Road, Oxford OX1 3TA, United Kingdom; ‡ Western Australian School of Mines, Curtin University, Bentley 6102, Australia; § Institute for Optics and Atomic Physics, 26524 Technische Universität Berlin, Hardenbergstrasse 36, Berlin 10623, Germany; ∥ Fritz-Haber-Institut der Max-Planck-Gesellschaft, Berlin 14195, Germany; ⊥ 6029 HFML-FELIX, Toernooiveld 7, Nijmegen 6525 ED, the Netherlands; # Institute for Molecules and Materials, Radboud University, Heyendaalseweg 135, Nijmegen 6525 AJ, the Netherlands

## Abstract

Carbon dioxide (CO_2_) fixation and subsequent activation represent grand challenges in materials science and chemical catalysis, with the aim of mitigating the worst impacts of global warming and associated climate change. Vibrational spectroscopy can provide essential structural insights into the binding and activation of CO_2_ on potential reduction catalysts. The nature of carbon dioxide adsorption on rhodium cluster anions, Rh_*n*_^–^ (*n* = 3–12), has been investigated using a combination of infrared free-electron laser spectroscopy and quantum chemical calculations. A clear cluster-size dependence to the nature of the binding is observed. On the smallest clusters, Rh_*n*_^–^ (*n* ≤ 4), CO_2_ is dissociatively adsorbed, as indicated by a carbonyl stretch at 1880 cm^–1^. By contrast, larger clusters, Rh_*n*_^–^ (*n* ≥ 7), exhibit highly activated molecular binding, but both motifs are observed on intermediate cluster sizes (*n* = 5, 6). The extent of chemical activation is clearly discernible spectroscopically and arises from the propensity of CO_2_ to attach across Rh–Rh bridge sites, coupled with significant electron transfer from the metal cluster.

## Graphical abstract

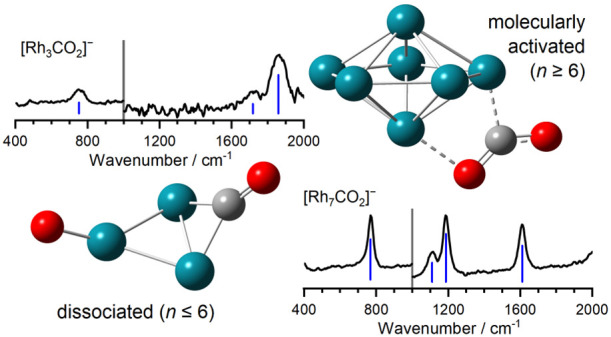

## Introduction

With average atmospheric carbon dioxide levels now well above 420 ppm,^1^ few global challenges are more significant than developing our understanding of the chemical activation and/or sequestration of this famously inert molecule. The prospect of using CO_2_ as a cheap and plentiful C1 feedstock for synthetic fuel production is highly appealing,^2,3^ but the kinetic and thermodynamic challenges are considerable as most reactions involve formidable activation barriers.^4–6^

Late transition metals, especially in highly divided form, are synonymous with heterogeneous catalysis, and the potential for CO_2_ activation at single crystal surfaces has been explored previously.^7–9^ Notably, the Rh(111) rhodium surface is believed to dissociate CO_2_ molecules at room temperature.^10^ Some of the most detailed insights into the key interactions involved, however, come from gas-phase studies, free from the complexity of solvents and supports.^11^ The simplest examples of metal-CO_2_ binding involve gas-phase M^±^(CO_2_)_*m*_ ion–molecule complexes. Infrared spectroscopy of M^+^(CO_2_)_*m*_ species has revealed linear, M^+^–O=C=O, binding, typically with weak molecular perturbation,^12–18^ though lanthanide and actinide oxide ions prove exceptions.^19–21^ By contrast, different M^–^(CO_2_)_*m*_ complexes exhibit a wide range of binding motifs reflecting differing, often extensive, CO_2_ activation,^22–28^ as reviewed comprehensively by Weber.^29^

The enhanced ability of anions to activate CO_2_ arises from efficient electron donation into the antibonding 2π_u_ LUMOs which destabilizes the molecule driving distortion from linearity; in turn, providing a spectroscopic signature of activation.^29,30^ For reference, full electron transfer to isolated CO_2_ leads to the thermodynamically unstable CO_2_^–^ anion which decays by electron emission;^31,32^ the metastable radical anion has been observed by photoelectron spectroscopy,^33^ but its structural (vibrational) properties are known best from matrix isolation studies.^34^

Atomically precise gas-phase metal clusters offer the intriguing potential of tuning the degree of molecular activation through their cluster size.^35,36^ These species represent simplified models for the defect sites of solid surfaces at which interesting chemistry occurs.^37^ Metal clusters are experimentally tractable and amenable to highly sensitive mass spectrometric detection, facilitating action spectroscopies such as infrared multiple photon dissociation (IR-MPD) spectroscopy.^38^ IR-MPD spectroscopy has been employed to study CO_2_ binding to Mn_*n*_O_*m*_^+^,^39^ Cu_*n*_^+^,^40^ Cu_*n*_C_*m*_^–^,^41^ Cu_*n*_O_*m*_^±^,^42^ Pt_*n*_O_*m*_^+^,^43^ Co_*n*_^–^,^44^ and Pt_*n*_^–^.^45^ Key spectral signatures of CO_2_ activation include a characteristic red-shift of the antisymmetric stretch (reflecting charge transfer), and developing infrared activity in the symmetric stretch, reflecting nonlinear CO_2_.^29,46^ Pertinent to this study, CO_2_ was found to adsorb only to larger cobalt cluster anions, Co_*n*_^–^ (*n* ≥ 7), and to dissociate in the process, as indicated by a strong metal–carbonyl, IR band at ∼1900 cm^–1^.^44^ Conversely, size-selective CO_2_ activation was observed on platinum cluster anions, Pt_*n*_^–^, with CO_2_ binding molecularly (but highly activated) on Pt_4_^–^ and dissociating on larger clusters.^45^ Although a key step in the reverse water gas-shift reaction, in many cases, CO_2_ dissociation may not be the optimal outcome, as strongly bound oxides and carbonyl species can lead to catalyst poisoning and reduced turnover.

Rhodium metal is well-known as a catalyst,^47^ famous for its role in the reduction of nitrogen oxides in automobile catalytic converters.^48^ Reflecting this, the structures^49–53^ and reactivity^51,54–56^ of rhodium clusters have been the subject of extensive research. Molecular binding motifs of NO_*x*_ to rhodium clusters have been investigated spectroscopically,^57–59^ and IR-driven bond-breaking chemistry leading to N_2_ loss observed for Rh_*n*_N_2_O^+^ clusters.^60–62^ Methane undergoes size-dependent reactivity with rhodium cluster anions, with Rh_3_^–^ and Rh_4_^–^ particularly active.^63,64^ Rhodium cluster anions have also been identified as efficient potential catalysts for the reverse water gas shift reaction, in mass spectrometric studies of reactions with CO_2_ and H_2_.^65,66^

Here, we present an infrared spectroscopic investigation of CO_2_ activation on gas-phase rhodium cluster anions, Rh_*n*_^–^ (*n* = 3–12), performed using the Free-Electron Laser for Intracavity Experiments (FELICE) at HFML-FELIX in Nijmegen, The Netherlands. Structural assignments are confirmed by comparison with simulated spectra of energetically low-lying isomers, calculated using density functional theory.

## Results and Discussion

Figure 1 shows a representative mass spectrum of the cluster distribution generated in the laser ablation source,^67^ with assignments based on empirical formulas. The mass spectrum is dominated by pure rhodium cluster anions, Rh_*n*_^–^, and [Rh_*n*_CO_2_]^−^ species with smaller oxide signals. We adopt the square bracket notation to indicate empirical formulas only. Examination of the mass spectrometric intensity ratios [Rh_*n*_(CO_2_)_*j*_]^−^:Rh_*n*_^–^ from Figure 1a,b reveal markedly different reactivity of each cluster size toward CO_2_. This is quantified in Figure 1c in terms of the relative effective rate constant, *k*_rel,*n*_, defined as

1 $$k_{\mathrm{rel},n}=\frac{k_{n}}{k_{n=3}}$$

and

**Figure 1:**
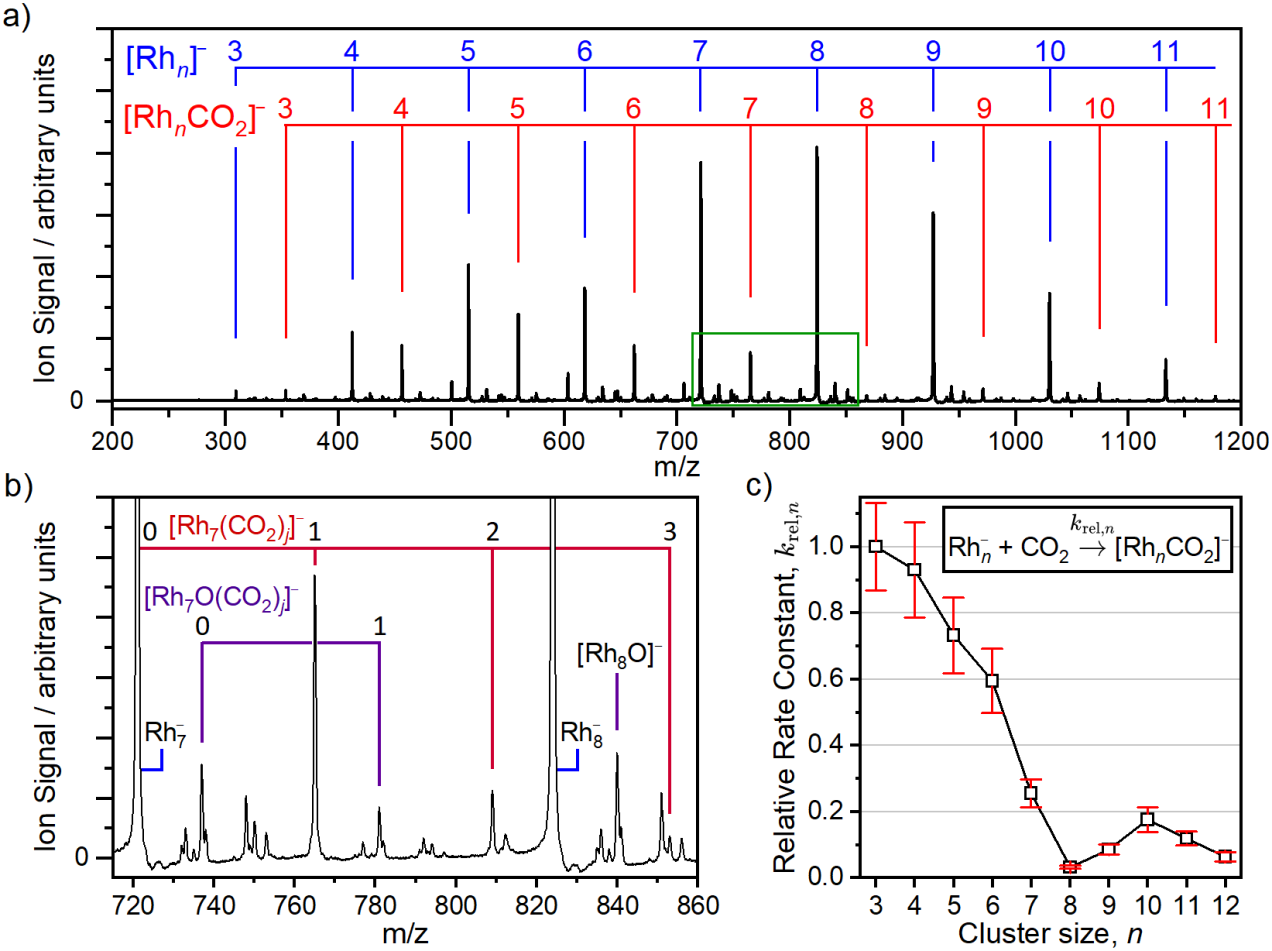
a) Representative mass spectrum of the anionic cluster distribution produced by ablation of a rhodium rod in a carrier gas of He, with CO_2_ introduced into the cluster channel downstream from the ablation point. b) Detailed view of *m/z* = 715–860 with additional assignments. c) Relative rate constants for CO_2_ binding on the rhodium cluster anions as a function of cluster size (for more kinetic analysis see Supporting Information).

$$k_{n}=\frac{RT}{p\left({\mathrm{CO}}_{2}\right)}\times \frac{1}{t}\times \ln\left(\frac{\left[R{h_{n}}^{-}\right]}{\overset{j_{\max}}{\underset{j=0}{\sum }}\left[[{\mathrm{Rh}}_{n}{({\mathrm{CO}}_{2}{)}_{j}]}^{-}\right]}\right)$$ 2

In this way, *k*_rel,*n*_ is independent of the CO_2_ partial pressure, *p*(CO_2_), temperature, *T*, and the effective reaction time, *t,* and can be calculated from integrated ion signals, [X^–^], extracted from the mass spectrum. The trend in reactivity with cluster size (Figure 1c) is a near linear decrease in CO_2_ adsorption (or CO_2_ sticking coefficient) with increasing *n*, upon which a pronounced dip in reactivity for *n* = 7–9 is superimposed, in good qualitative agreement with the ion trap experiments of Liu et al. (see Supporting Information for direct comparison).^66^ A Hirshfeld charge analysis of calculated Rh_*n*_^–^ structures suggests cluster charge dilution as a major driving force of CO_2_ sticking (see Section 2, Supporting Information).

Figure 2 presents the infrared action spectra of [Rh_*n*_CO_2_]^−^ (*n* = 3–12) clusters between 400 and 2000 cm^–1^, recorded in separate scans of 400–1000 cm^–1^ and 1000–2000 cm^–1^. Each spectral band observed results from resonant infrared absorption leading to loss of the precursor ion. For reasons that will become clear, we believe the dominant loss mechanism to be infrared multiple photon dissociation (IR-MPD) but we cannot exclude the possibility of resonant electron photodetachment. In Figure 2, the colored boxes indicate infrared bands characteristic of distinct structural motifs by which the spectra can be grouped. The smallest clusters, [Rh_*n*_CO_2_]^−^ (*n* = 3, 4), exhibit broad spectral features around 770 cm^–1^ (purple) and 1880 cm^–1^ (orange) only. By contrast, all [Rh_*n*_CO_2_]^−^ (*n* ≥ 7) spectra have bands around 780 cm^–1^ (red), 1150 cm^–1^ (green) and 1630 cm^–1^ (blue). The spectra of intermediate cluster sizes, [Rh_*n*_CO_2_]^−^ (*n* = 5, 6), exhibit bands in all four spectral regions.

**Figure 2:**
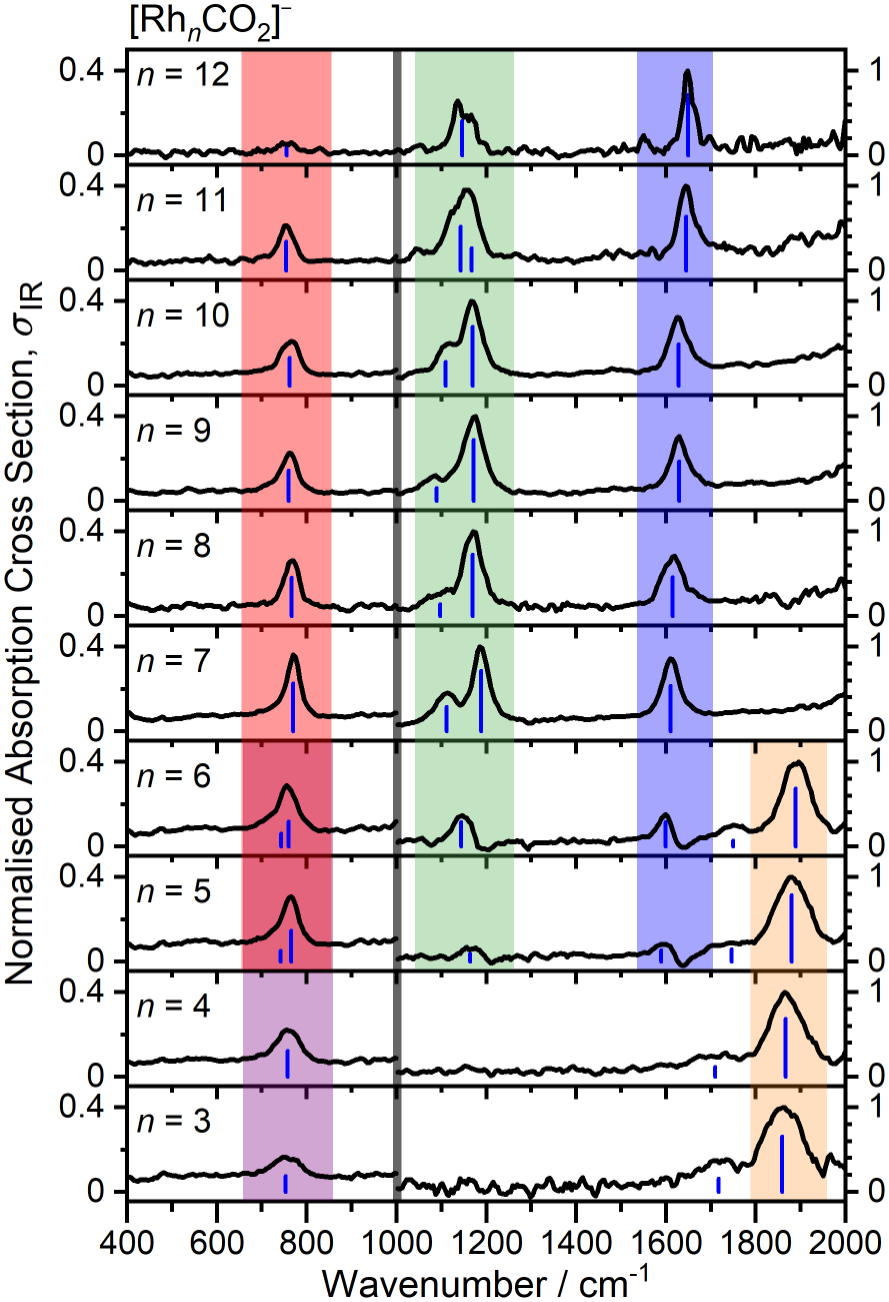
Normalized infrared action spectra of [Rh*_n_* CO_2_]^−^ (*n* = 3–12) clusters showing bands characteristic of (i) dissociative adsorption (orange and purple), and (ii) molecular, highly activated binding (blue, green, and red) of CO_2_. The gray vertical line separates the two distinct wavenumber regions over which data was recorded (400–1000 cm^–1^ and 1000–2000 cm^–1^). Note the difference in ordinate scales for the two regions. Blue lines mark experimental band centers.

The infrared spectra are readily interpreted in terms of changes in the nature of the CO_2_ binding with cluster size. The three bands in the spectra associated with the larger clusters, [Rh_*n*_CO_2_]^−^ (*n* ≥ 7), signify molecular, highly activated CO_2_, with distinct bend (770 cm^–1^), symmetric stretch (1150 cm^–1^) and antisymmetric stretch (1590–1650 cm^–1^) fundamental transitions.^45^ For the smaller clusters (*n* ≤ 6), the band at 1880 cm^–1^ is characteristic of a carbonyl C=O stretch, and is thus indicative of CO_2_ dissociation on these cluster anions.^68,69^ The metal-bound carbonyl bands observed here are in good agreement with those reported by Fielicke et al. in their IR-MPD study of pure Rh_*n*_CO^+/0/–^ clusters and are confidently assigned as mainly atop bound (μ_1_)-CO, accompanied by weaker red-shifted shoulders indicating bridged (μ_2_)-CO binding.^69^

The lowest energy spectral features at 700–800 cm^–1^ in Figure 2 arise from the IR-active CO_2_ bend or a Rh–O stretching vibration, or both, depending on the cluster size.^52,53^ The spectra associated with [Rh_5,6_CO_2_]^−^ indicate both CO_2_ and CO modes, suggesting a combination of molecular and dissociative CO_2_ binding. Minor dips in these spectra of [Rh_5,6_CO_2_]^−^ (*e.g.,* 1200 cm^–1^ and 1650 cm^–1^) match strong absorption bands in [Rh_5,6_(CO_2_)_2_]^−^ species also present (see below) which leads to weak enhancements in the spectra of the product species. Overall, the infrared spectra indicate a clear size-selective activation of the CO_2_ molecules.

In order to verify the assignments of the spectral bands observed, the experimental IR-MPD spectra can be compared with simulated spectra of low energy structures calculated using density functional theory (DFT) at the UB3P86/def2-TZVPPD level. Figure 3 shows such a comparison for the [Rh_3_CO_2_]^−^ and [Rh_7_CO_2_]^−^ clusters, and similar comparisons for [Rh_*n*_CO_2_]^−^ (*n* = 4–6) are included in the Supporting Information. The structures and simulated spectra of the calculated global minimum structure as well as two energetically low-lying structures are shown. For each cluster size, both molecularly and dissociatively bound structures are included for completeness, and are similar to those reported previously utilizing the PBE pure functional.^66^

**Figure 3:**
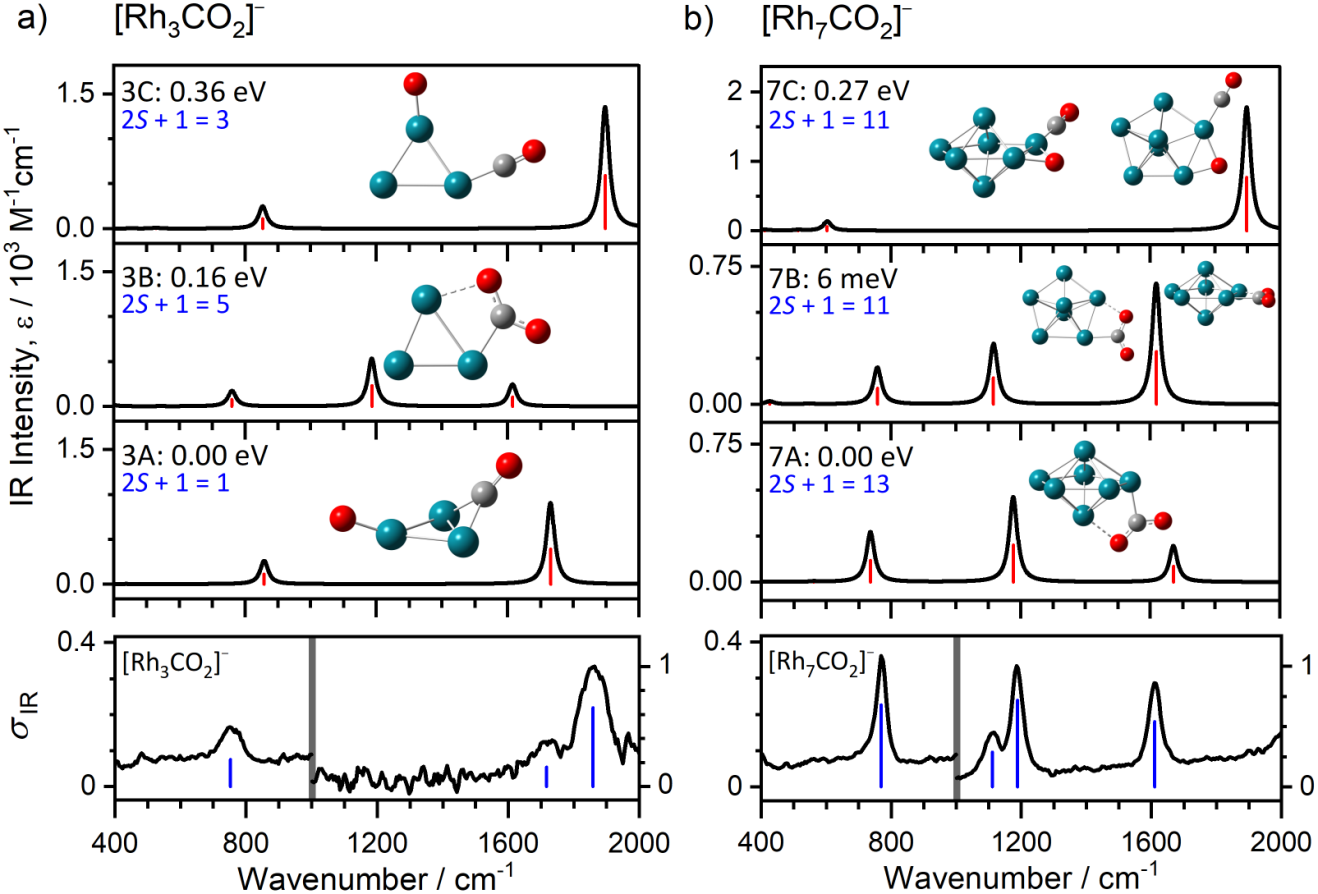
Comparison of experimental and UB3P86/def2-TZVPPD simulated IR spectra for [Rh*_n_* CO_2_]^−^ for a) *n* = 3 and b) *n* = 7. The relative energies and spin multiplicities of different isomers are given. Detailed structures and harmonic frequencies of these and other cluster sizes are given in the Supporting Information. Calculated vibrational bands (red lines) have been convoluted with Lorentzian line shapes of 30 cm^–1^ fwhm for ease of comparison. Blue lines mark experimental line centers.

The experimental [Rh_3_CO_2_]^−^ spectrum exhibits features assignable to a combination of atop (μ_1_) bound Rh–O, as well as μ_1_- and μ_2_-bound CO (i.e., Isomers 3A, 3C) confirming dissociative adsorption. We find no evidence of molecularly bound structures, such as Isomer 3B, since no bands in the region of the symmetric CO_2_ stretch around 1200 cm^–1^ are observed.

The [Rh_7_CO_2_]^−^ spectrum (Figure 3b) is qualitatively different to that of [Rh_3_CO_2_]^−^, and is readily assigned as a molecularly bound, highly activated (μ_2_, η^2^)-CO_2_ unit, resulting in clear bending, symmetric stretch and antisymmetric stretch modes. The bands at 1110 cm^–1^ and 1190 cm^–1^ are assigned to CO_2_ symmetric stretches on different, near isoenergetic, isomeric forms of the cluster. In one case (Isomer 7B), CO_2_ is bound μ_2_ across a Rh–Rh bond *within* the Rh_5_ plane (σ_*h*_) of the Rh_7_^–^ pentagonal bipyramid structure, and in the other (Isomer 7A), between the Rh_5_ plane and one of the apex Rh atoms. The degree of CO_2_ activation arising from charge transfer is apparent in the large red-shift of the antisymmetric stretch fundamental by approximately 700 cm^–1^, from 2349 cm^–1^ in free CO_2_,^70^ to 1630 cm^–1^ – close to that of the radical anion (CO_2_^•–^, 1658 cm^–1^).^34^ According to Weber’s systematic analysis of [MCO_2_]^−^ complexes, this red-shift is consistent with near-complete electron transfer.^29^ The activated CO_2_ molecule on [Rh_7_CO_2_]^−^ is significantly bent, with a calculated bond angle of 135°, and the C=O bond lengths are distorted from 1.16 Å in free CO_2_, to 1.23 Å and 1.25 Å. Clearly, binding to a metal cluster changes the effective reduced mass as well as the force constant,^45^ but even so, the large red-shift and the bond elongation confirm significant charge transfer from the metal cluster to the LUMO of CO_2_.

Further confirmation of the changing nature of the CO_2_ binding comes from matching depletions in the precursor ion [Rh_*n*_CO_2_]^−^ spectra with corresponding enhancements in plausible product species: [Rh_*n*_O]^−^ (corresponding to CO loss) or Rh_*n*_^–^ (full CO_2_ loss). Figure 4 shows such a comparison for [Rh_6_CO_2_]^−^ and [Rh_7_CO_2_]^−^. For [Rh_7_CO_2_]^−^ (Figure 4b), each absorption band in the precursor ion is reproduced, almost exclusively, as an enhancement in the bare Rh_7_^–^ signal, due to molecular CO_2_ loss. The mixed dissociative and molecular binding of CO_2_ in [Rh_6_CO_2_]^−^ is also reflected in Figure 4a with bands assigned to molecularly–bound isomers reproduced in enhancements in the Rh_6_^–^ product (arising from CO_2_ loss) and absorptions in dissociatively–bound species only producing Rh_6_O^–^ (CO loss). Weak production of Rh_6_O^–^ from bands associated with molecular binding are likely due to IR-induced CO_2_ dissociation competing with CO_2_ desorption, or else from CO_2_ loss from [Rh_6_OCO_2_]^−^. In all cases, these different fragmentation spectra confirm the assignments above.

**Figure 4:**
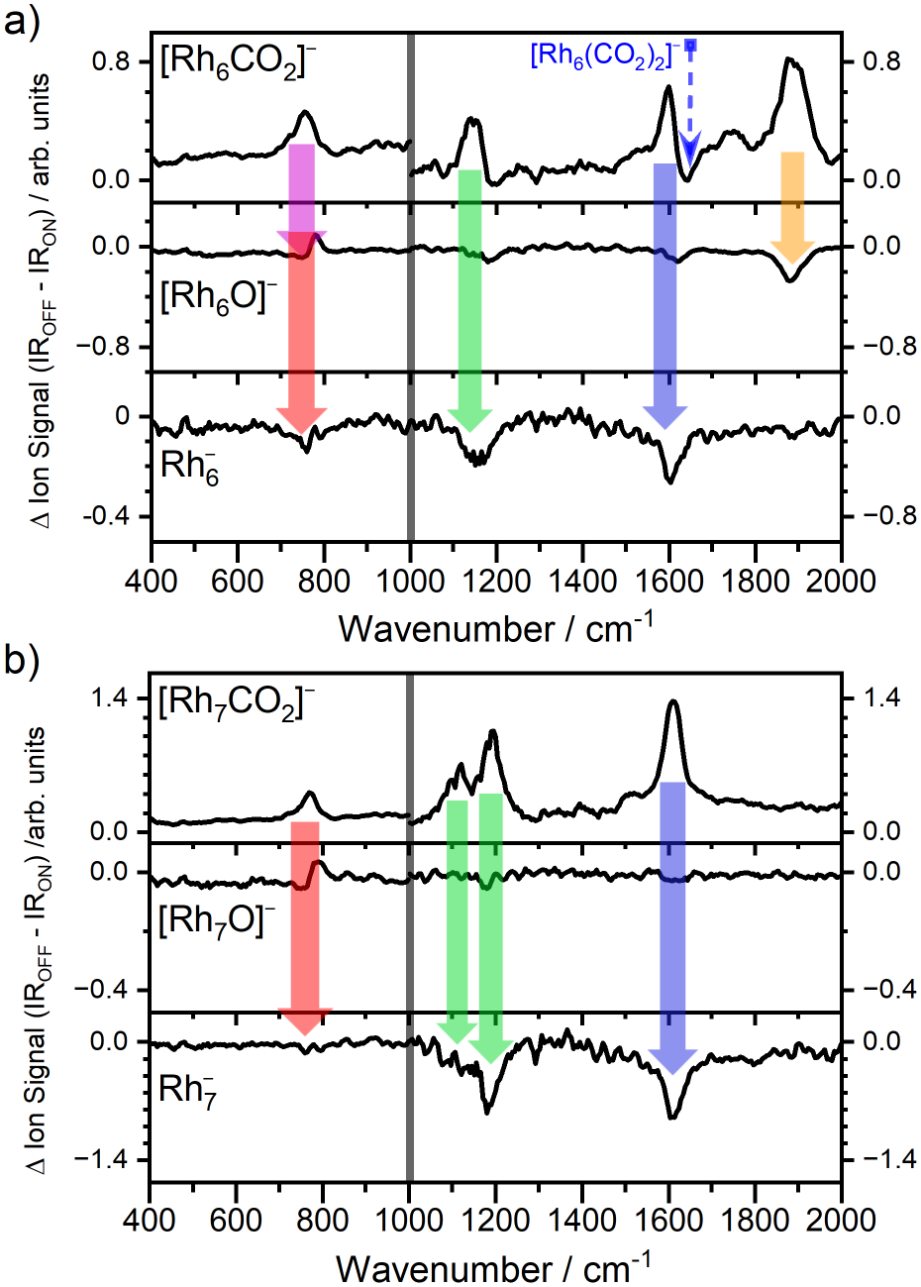
IR–MPD spectra of a) [Rh_6_CO_2_]^−^ and b) [Rh_7_CO_2_]^−^ along with spectra recorded simultaneously in the potential product fragment channels [Rh*_n_* O]^−^ (corresponding to CO loss) and Rh*_n_* ^–^ (CO_2_ loss).

The close agreement of experimental and simulated spectra, as well as IR-dependent fragmentation, provides considerable support for the assignments of size-selective, reductive activation of CO_2_ on the clusters. Dissociative adsorption is found on the smallest clusters studied (*n* = 3, 4), whereas an increasing propensity toward (activated) molecular binding is found for larger clusters. Importantly, the binding of CO_2_ is exclusively molecular on clusters *n* ≥ 7. It is noteworthy that this trend mirrors that of the reaction rate constants in Figure 1c, with the most reactive clusters leading to CO_2_ dissociation. The switch to molecular binding is commensurate with the fall in reactivity between *n* = 5 and 8. These observations reflect trends in the adiabatic detachment energy (electron binding energy) of bare Rh_*n*_^–^ (*n* = 1–9) determined from photoelectron spectroscopy by Bowen and coworkers, which showed step increases between *n* = 4 (0.7 eV) and 5 (1.4 eV) and again between *n* = 6 (1.4 eV) and 7 (2.1 eV), beyond which they remain near constant.^71^ Perhaps unsurprisingly, increased electron binding energy reduces the ability of a cluster to reduce the CO_2_ moiety.

It is worth emphasizing quite how different the binding of CO_2_ on rhodium cluster anions is from that we have reported previously on cobalt and platinum cluster anions.^44,45^ CO_2_ was found to bind only on larger Co_*n*_^–^ clusters (*n* ≥ 7) and, even then, exclusively dissociatively. On Pt_*n*_^–^, CO_2_ is molecularly activated on *n* = 4 but dissociates on *n* = 5, 6, and 7, the reverse trend to that observed for Rh_*n*_^–^. It is clear that more studies are required before any pattern emerges for carbon dioxide activation on late transition metal clusters.

The transition from dissociative to molecularly activated CO_2_ binding observed in the current study, and the coexistence of both motifs on some cluster sizes, warrants investigation of the reactive potential energy surface. Figure 5 shows such a surface for the Rh_3_^–^ + CO_2_ reaction calculated at the CCSD­(T)/aug-cc-pVDZ//UB3P86/def2-TZVPPD level of theory. That is, for geometries optimized at the DFT level, single-point energies are recalculated with CCSD­(T)/aug-cc-pVDZ. In this way we seek to mitigate the known deficiencies of DFT in calculating transition states. Beyond the initial entrance channel complex, the key minima include the lowest energy molecularly bound (Mol_MIN_) and dissociatively bound (Diss_MIN_) structures. The associated transition states (‡) were located using the synchronous transit-guided quasi-Newton method and confirmed by intrinsic reaction coordinate calculations. Of particular relevance to the current results, the transition state for CO_2_ dissociation (i.e., TS2) is calculated to be submerged on only the singlet surface, suggesting spin-crossover dynamics from the ground state quintet ^5^Rh_3_^–^ + CO_2_ surface. Although we cannot be certain of the electronic states produced in the cluster source, it seems that the propensity for CO_2_ to dissociate on smaller rhodium cluster anions is driven by nonadiabatic dynamics. Previous DFT calculations found the barrier on the quintet surface to be submerged;^66^ however, transition state energies do represent a challenge to DFT methods.

**Figure 5:**
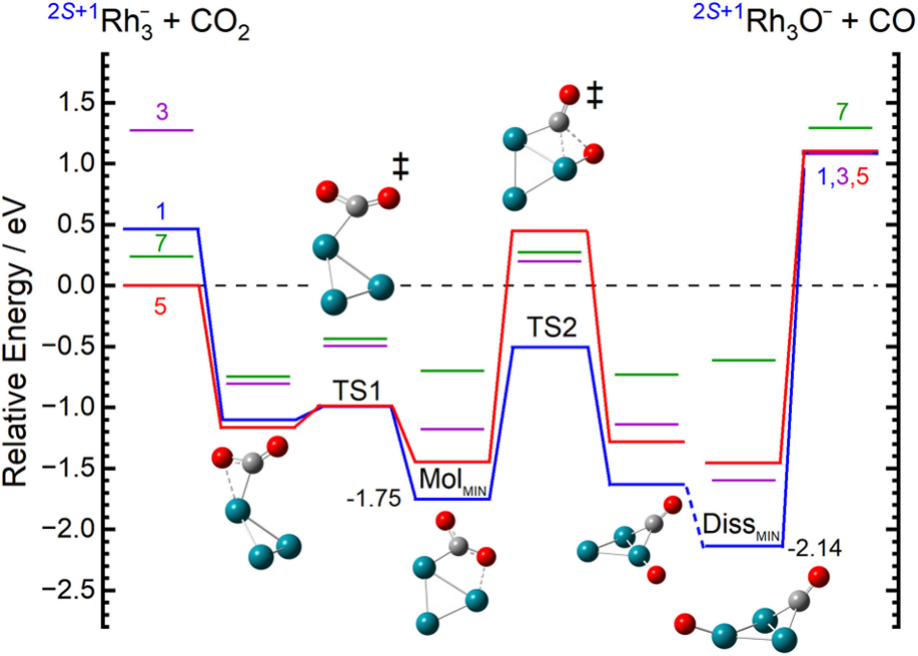
Truncated CCSD­(T)/aug-cc-pVDZ//UB3P86/def2-TZVPPD potential energy surface of Rh_3_^–^ + CO_2_ along the molecular binding and dissociative coordinates. The most pertinent surfaces are those of the singlet (blue) and quintet (red) spin states: stationary point energies are also indicated for the triplet (purple) and septet (green) states. Key transition states (‡) and the most stable structures of [Rh_3_(CO_2_)]^−^ (Mol_MIN_) and [Rh_3_O­(CO)]^−^ (Diss_MIN_) are shown. Detailed structures and harmonic frequencies are provided in Tables S11–S15, Supporting Information.

The observed trend toward molecular activation on larger clusters appears to be driven by thermodynamics with the Mol_MIN_ structures falling ever lower in energy relative to the Diss_MIN_ with increasing cluster size (cf. Table S15 in Supporting Information). This spectroscopic study, with its unambiguous identification of the CO_2_ binding motif, is thus consistent with the interpretation of the mass spectrometric reactivity data from He and coworkers.^66^ A detailed, high-level computational study of the reactive potential energy surfaces for larger clusters would attract great interest, but remains beyond the scope of the current experimental study.

At higher CO_2_ partial pressures, sufficient [Rh_*n*_(CO_2_)_2,3_]^−^ clusters were produced to record their infrared spectra, as shown in Figure 6, from which information on the activation of additional adsorbed molecules can be deduced. Rh_*n*_^–^ (*n* = 3, 4) clusters appear capable of dissociating a second CO_2_ molecule, as indicated by the intense carbonyl bands at 1800–2000 cm^–1^ in conjunction with the lack of molecular CO_2_ bands (Figure 6a). However, for *n* = 4, there is evidence that the third CO_2_ binds molecularly with the appearance of new bands at *ca*. 1190 cm^–1^ and 1670 cm^–1^ (Figure 6b). In contrast to the spectra of the single-adsorbate clusters, the IR-MPD spectra for [Rh_*n*_(CO_2_)_2,3_]^−^ (*n* = 5, 6) are now dominated by activated, molecularly bound CO_2_, as only weak carbonyl bands at 1900 cm^–1^ are recorded. This strongly suggests that not only do the second and third CO_2_ bind without triggering dissociation of previously bound CO_2_ molecules, but that the charge remaining on the metal cluster in [Rh_5,6_CO_2_]^−^ is insufficient to reductively activate CO_2_ to the point of dissociation.

**Figure 6:**
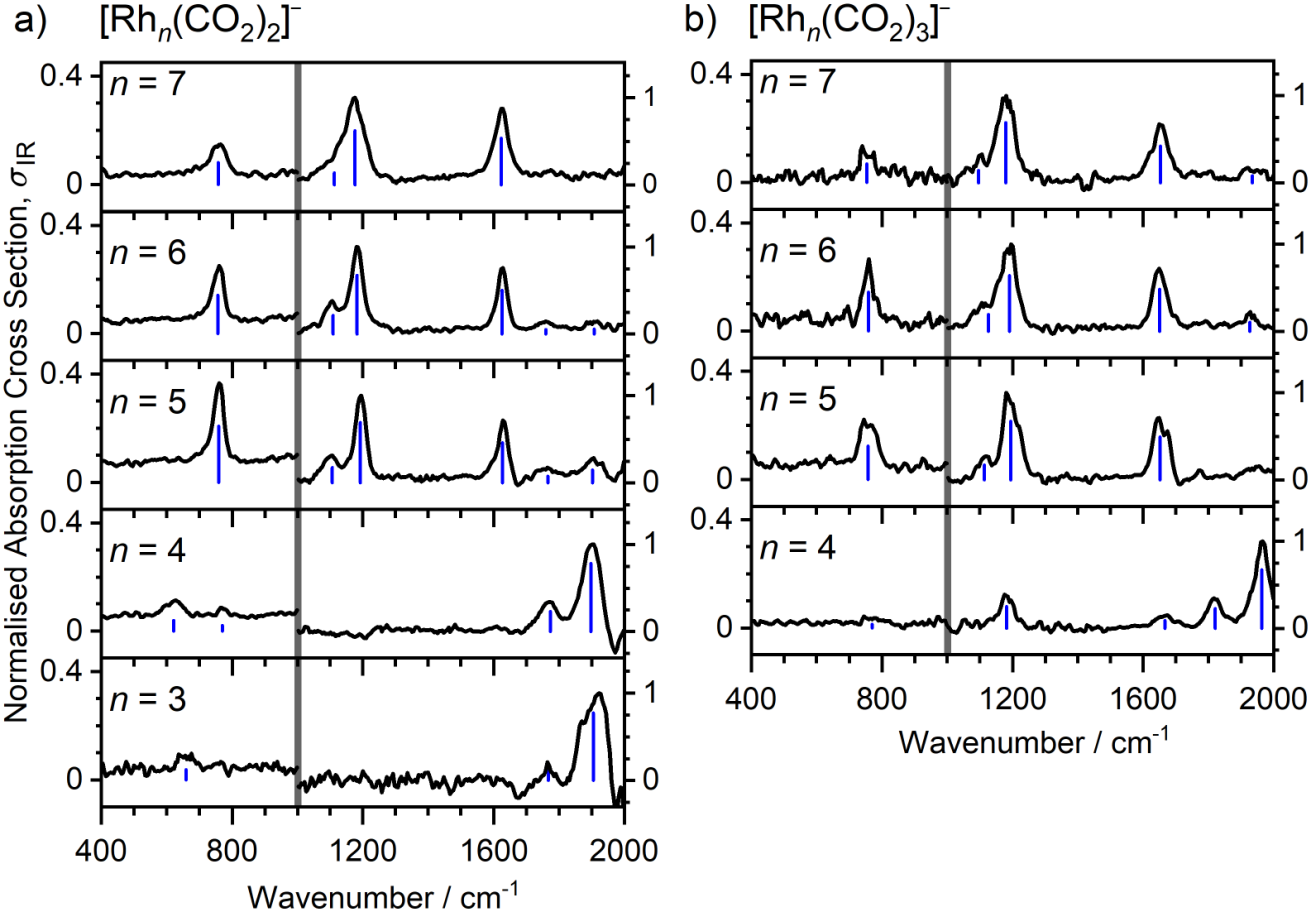
Normalized IR-MPD spectra of a) [Rh*_n_* (CO_2_)_2_]^−^ (*n* = 3–7) and b) [Rh*_n_* (CO_2_)_3_]^−^ (*n* = 4–7), showing multiple molecularly bound CO_2_ ligands (*n* = 5, 6, and 7), and dissociative binding on clusters *n* = 3 and 4. Note the difference in ordinate scales for the two spectral regions.

The spectra of the [Rh_7_(CO_2_)_2,3_]^−^ clusters are similar to that of [Rh_7_(CO_2_)]^−^, suggesting similar, molecular, binding. Rh_7_^–^ is therefore capable of activating at least three CO_2_ molecules simultaneously and, indeed, represents an excellent target for chemical reactivity of activated CO_2_.

## Conclusions

In summary, IR-MPD spectroscopy of CO_2_ adsorption to rhodium cluster anions has revealed a clear cluster-size dependence to the degree of activation. Dissociative adsorption – characterized by clear μ_1,2_ C=O carbonyl stretches – dominates binding on the smaller clusters (*n* ≤ 6). Highly activated, molecular binding is observed on larger clusters (*n* ≥ 5), and this is the only motif observed for *n* ≥ 7. Given the charge transfer mechanism of CO_2_ activation, these results suggest larger Rh_*n*_^–^ clusters are weaker reductants, consistent with a charge dilution model (cf. diminishing red-shift in CO_2_ antisymmetric stretch with increasing cluster size, *n*). The degree of this reductive activation mirrors trends observed in the relative rates of reaction associated with CO_2_ binding on the rhodium cluster anions, as well as the electron detachment energies of the anion clusters. Structural assignment is confirmed by DFT simulations, with calculated spectra, reproducing the experimentally observed spectral features well. The trends presented here are in marked contrast to previous studies of CO_2_ binding to transition metal clusters, in which only dissociative binding to Co_*n*_^–^ was observed,^44^ and molecular binding was observed on only the smallest Pt_*n*_^–^ clusters.^45^

The cluster size dependence to the degree of CO_2_ activation reported along with the binding motifs themselves, cluster geometrical structures and the role of charge dissipation all represent significant implications for the design of practical rhodium-based catalysts for CO_2_ fixation and/or reactivity. The importance of Rh–Rh bridge sites in the activation step and local charge fixation suggest that fine control over the degree of activation (versus full dissociation) might be achieved by doping pure clusters with atoms of other elements. In this way it is hoped that this study will provide inspiration for the use of small, redox-active, transition metal clusters in the design of novel CO_2_ fixation materials, with the intention of enhancing further chemical reactivity in a catalytic manner.

## Experimental and Computational Methods

The instrument and FELICE beamline have been described in detail previously.^67,72^ In brief, rhodium anion clusters were produced by pulsed laser ablation of a rotating and translating rhodium rod. CO_2_ was introduced through a secondary valve in the cluster channel downstream, and the resultant anionic cluster production was probed by reflectron time-of-flight mass spectrometry. The source operates at 20 Hz and IR-MPD spectra were recorded by exposing alternate cluster pulses to the 10 Hz FELICE beam in the spectral range 400–2000 cm^–1^. Spectra were recorded as depletions of the precursor cluster ion signals and are presented as normalized absorption cross sections, σ_IR_, generated at a particular FEL wavelength according to

3 $$\sigma_{\mathrm{IR}}=-\frac{1}{\phi \left(\lambda \right)}\ln\left(\frac{I_{\mathrm{ON}}}{I_{\mathrm{OFF}}}\right)$$

where *I*_ON_ and *I*_OFF_ are the FEL-on and FEL-off ion signals respectively, and ϕ­(λ) is the wavelength-dependent, intracavity, FELICE macropulse energy (Supporting Information for more details).

Assignment of the experimental spectra has been aided by comparison with simulated IR spectra of energetically low-lying isomers calculated with DFT utilizing the Gaussian 16 software package.^73^ The UB3P86 ^74,75^ /def2-TZVPPD^76–78^ level of theory has been used given the performance based on previous work with respect to metal cluster – ligand complexes.^79^ In addition to chemical intuition, a modified Kick^3^ algorithm was employed to generate starting geometries allowing broad searches of the various conformer spaces.^80,81^ The calculated vibrational fundamental bands have been scaled by a factor of 0.964: the anharmonic correction factor to the antisymmetric stretch of free CO_2_ at this level of theory.^70^ To investigate the reaction potential energy surface, the coupled cluster method with singles, doubles, and perturbative inclusion of triples (CCSD­(T)) was utilized, in conjunction with an aug-cc-pVDZ (−PP for Rh) basis set.^82–84^

## Supplementary Material



## Data Availability

The experimental data presented here are publicly available from the Oxford Research Archive (DOI: 10.5287/ora-erm7nq20a).

## Notes

Additional reactivity mass spectrometric data and comparisons; full computational results, including structures, geometry files, and simulated IR spectra (PDF)
