# Supplementary material for: Size-Selective CO2 Activation at Rhodium Cluster Anions
Source: J Am Chem Soc. 2026 Jul 1;148(27):28682–90. doi: 10.1021/jacs.6c05767 (PMC13383710; doi:10.1021/jacs.6c05767)
Supplement: Supplementary file 1 [file ja6c05767_si_001.pdf]

Size-Selective CO_2 Activation at Rhodium Cluster Anions

*Christian T. Haakansson,[1] David J. Vesty,[1] Peter T. Rubli,[1] Peter D. Watson,[1,2] Ellen A. Jones,[1] Jasmin P. Justen,[3] André Fielicke,[4] Joost M. Bakker,[5,6] and Stuart R. Mackenzie[1]**

[1] Department of Chemistry, University of Oxford, Chemistry Research Laboratory, Mansfield Road, Oxford OX1 3TA, United Kingdom

[2] Western Australian School of Mines, Curtin University, Bentley, 6102, Australia

[3] Institute for Optics and Atomic Physics, Technische Universität Berlin, Hardenbergstrasse 36, 10623 Berlin

[4] Fritz-Haber-Institut der Max-Planck-Gesellschaft, 14195 Berlin, Germany

[5] HFML-FELIX, Toernooiveld 7, Nijmegen 6525 ED, The Netherlands

[6] Institute for Molecules and Materials, Radboud University, Heyendaalseweg 135, Nijmegen 6525 AJ, The Netherlands

Supporting Information

The supporting information presented here comprises of additional reactivity data associated with the reactivity of CO_2 and Rh_n^- as well as results of DFT calculations performed on $[Rh_nCO_2]^-$ clusters calculated at the UB3P86/def2-TZVPPD level of theory. Information included are simulated spectra, harmonic frequencies, and cartesian coordinates of optimised structures. In addition, further energetics and structural information is provided with respect to the reaction potential energy surface of the dissociation of CO_2 on Rh_3^-. In addition to the computational data, an example raw signal, as well as the FELICE intracavity power curve, and resultant power-corrected absorption cross-section data has also been provided.

Contents

1. Comparison of rate constants from mass spectrometric data 3
2. Relation between rate constants and cluster charge 4
3. Further comparison of experimental and simulated spectra 5
4. Harmonic frequency analysis of calculated low-energy structures 8
5. Cartesian coordinates of calculated low-energy structures 20
6. Hamonic analysis and cartesian coordinates of key structures in the potential energy surface Figure 4........ 28
7. Data analysis, power correction and cross-section determination 35

1. Comparison of rate constants from mass spectrometric data

As noted in the main manuscript, relative rate constants have been extracted from the mass spectral data through integration of the individual mass peaks, the data of which can be seen in Figure S1 in black (SRM). In order to further corroborate this data, a comparison has been made to previously recorded mass spectral data shown in Figure S1 in red and blue.

The red dataset (AF) was recorded by co-authors AF and JPJ at the Fritz-Haber-Institute, Berlin, using a similar laser ablation cluster source described previously [N. X. Truong, M. Haertelt, B. K. A. Jaeger, S. Gewinner, W. Schöllkopf, A. Fielicke, O. Dopfer, *Int. J. Mass Spectrom.* **2016**, *395*, 1-6]. Briefly, a translating and rotating rhodium rod undergoes laser ablation, with reactant CO_2 gas introduced downstream in the reaction channel, following which the cluster distribution is mass spectrometrically probed by way of a time-of-flight reflectron mass spectrometer. Mass spectra were recorded at twenty different partial pressures of CO_2 introduced in the reaction channel and the integrals associated with each mass peak of the form $[Rh_n(CO_2)_j]^-$ for j = 0, 1, 2, 3 extracted. Plotting of $\ln([Rh_n^-]/\Sigma([Rh_n(CO_2)_j]^-)$ against $p(CO_2)$ then allows fitting in order to extract a rate constant.

The blue dataset (SG) has been provided courtesy of Prof. Sheng-Gui He and was published in a previous mass spectrometric study of Rh_n^- + CO_2 reactivity [Y-Z. Liu, X-Y. He, J-J. Chen, Z-P, Zhao, X-N. Li, S-G. He, *Datlon Trans.* **2023**, *52*, 6668-6676]. Overall, despite the marked differences in techniques employed (ion trap versus higher pressure reaction channel), the relative rate constants produced from each set of data agree well (Figure S1). The one notable discrepancy is the efficiency of adsorption on n = 3, the smallest cluster size studied. It seems likely that this difference arises due to the markedly different gas pressures used in the different experiments, with the higher pressures stabilising the adsorption on the smallest clusters.

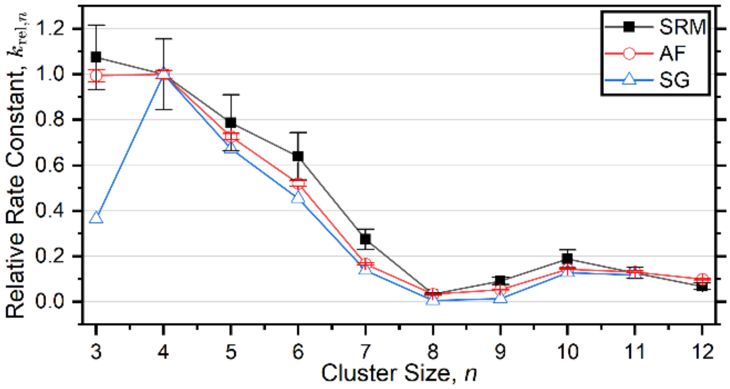

Figure S1: Relative rate constants associated with the reactivity of size-selected rhodium cluster anions with CO_2, with data shown in black presented in the main manuscript (Figure 1C), data shown in red extracted from separate mass spectral data collected at the Fritz-Haber-Institute, and data shown in blue provided by the authors of previous work.

2. Relation between rate constants and cluster charge

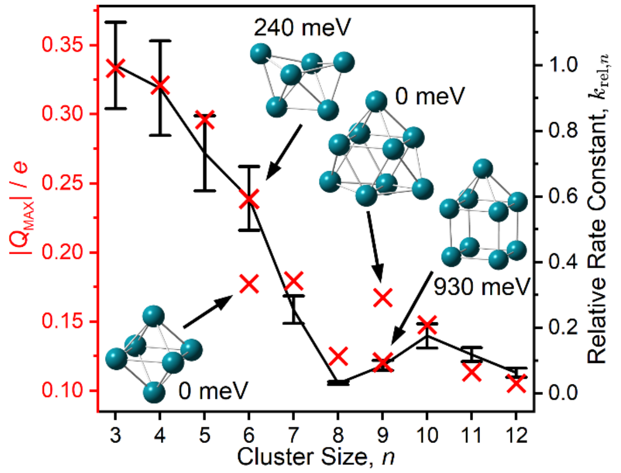

Figure S2. Relative rate constants, $k_{rel,n}$, for CO_2 binding on the rhodium cluster anions as a function of cluster size, overlayed with the magnitude of the maximum charged rhodium atom within the bare anion cluster. Deviations from this correlation between charge and $k_{rel,n}$ for n = 6 and 9 can be accounted for through energetically higher-lying isomers based on DFT calculations.

To further explore the size-dependence of the $Rh_n^- + CO_2$ reaction shown Figure 1c, we have undertaken a Hirshfeld charge analysis of low energy bare rhodium cluster anions. For clusters larger than n = 7, our starting structures were sourced from [T. D. Hang, H. M. Hung, L. N. Thiem, H. M. T. Nguyen, *Comput. Theor. Chem.* **2015**, *1068*, 30-41]. The results are shown in Figure S2 superimposed upon the relative rate constants. The close correlation supports the idea that the relative rate constant, $k_{rel,n}$, is directly related to the charge, Q_{max}, on the most negative rhodium atom in the cluster. In turn this suggests that charge dilution is a major driving force of the size-dependence of the sticking probability. The local minimum observed at n = 8 reflects the highly symmetrical near cubic structure of Rh_8^- (O_h), with even charge distribution across the cluster.

3. Further comparison of experimental and simulated spectra

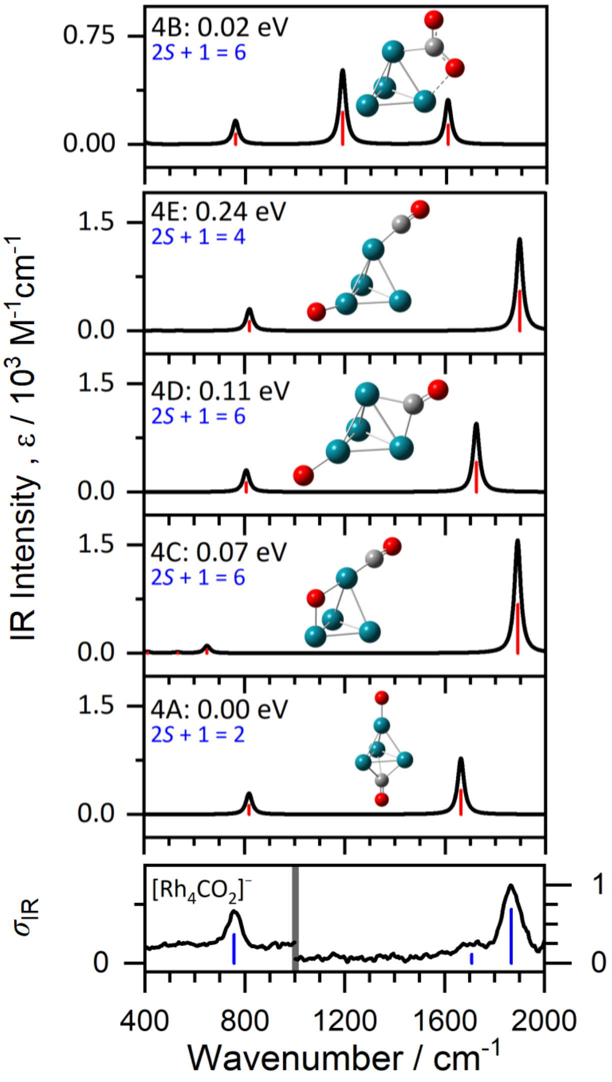

Figure S3: Comparison of experimental and simulated IR spectra for low-energy $[Rh_4CO_2]^-$ structures. In each case the energies relative to the global minimum have been provided as well as the spin multiplicities. Lorentzian fits of fwhm 30 cm^{-1} centred on the scaled calculated peaks are indicated with red lines, and the line centres of Gaussian fitted functions on the experimental spectra are shown with blue lines.

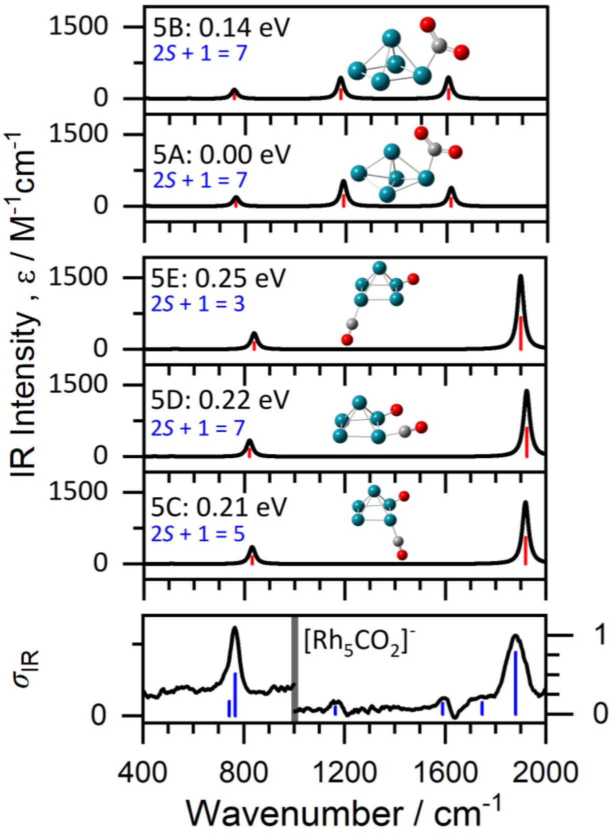

Figure S4: Comparison of experimental and simulated IR spectra for low-energy $[Rh_5CO_2]^-$ structures. In each case the energies relative to the global minimum have been provided as well as the spin multiplicities. Lorentzian fits of fwhm 30 cm^{-1} centred on the scaled calculated peaks are indicated with red lines, and the line centres of Gaussian fitted functions on the experimental spectra are shown with blue lines.

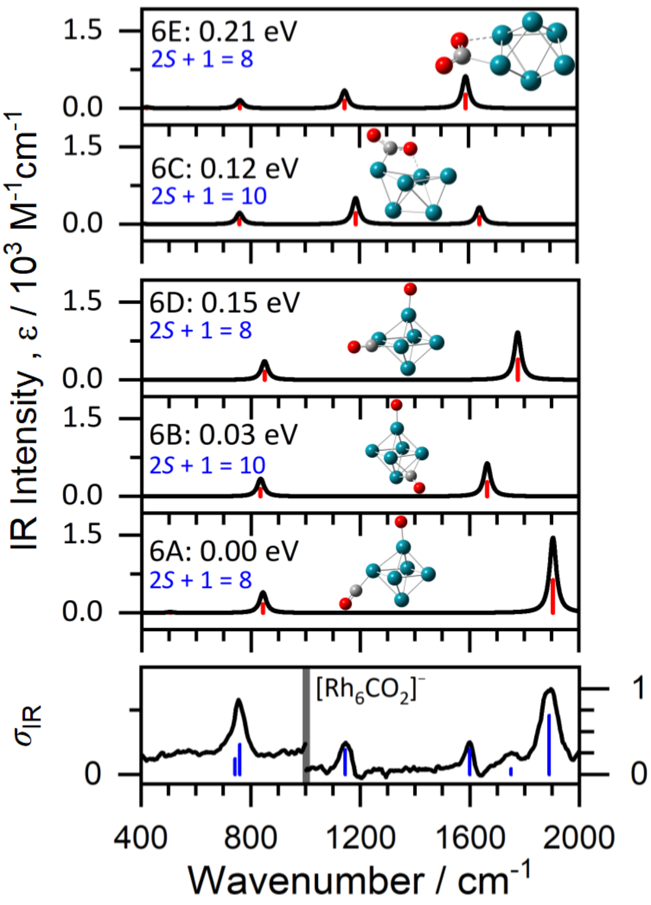

Figure S5: Comparison of experimental and simulated IR spectra for low-energy $[Rh_6CO_2]^-$ structures. In each case the energies relative to the global minimum have been provided as well as the spin multiplicities. Lorentzian fits of fwhm 30 cm^{-1} centred on the scaled calculated peaks are indicated with red lines, and the line centres of Gaussian fitted functions on the experimental spectra are shown with blue lines.

4. Harmonic frequency analysis of calculated low-energy structures

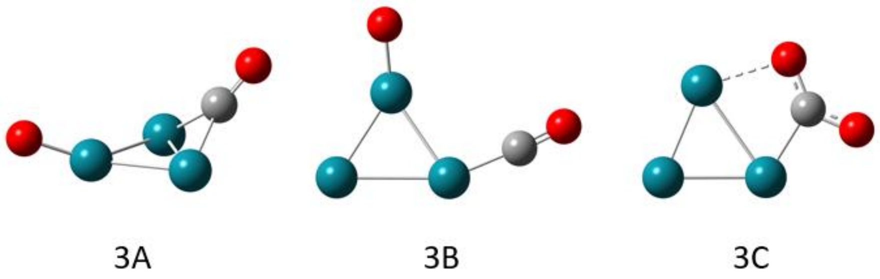

Table S1: Calculated harmonic frequencies of the $[Rh_3CO_2]^-$ structures displayed in Figure 3.

Structure	Mode	Frequency (cm^{-1})	Intensity (km/mol)
3A	ω_1	57.3	5.1
	ω_2	141.7	0.1
	ω_3	143.4	1.7
	ω_4	175.9	1.5
	ω_5	220.8	1.1
	ω_6	224.9	2.0
	ω_7	278.0	0.4
	ω_8	454.4	1.9
	ω_9	484.6	0.4
	ω_{10}	552.6	0.3
	ω_{11}	890.7	282.4
	ω_{12}	1795.7	982.0
3B	ω_1	24.7	2.3
	ω_2	106.2	0.1
	ω_3	146.2	1.0
	ω_4	170.6	1.4
	ω_5	208.2	0.8
	ω_6	241.8	15.7
	ω_7	279.1	1.5
	ω_8	402.2	32.1
	ω_9	562.4	1.5
	ω_{10}	788.8	187.1
	ω_{11}	1230.8	575.0
	ω_{12}	1675.4	264.6
3C	ω_1	44.4	0.2
	ω_2	67.3	0.5
	ω_3	110.6	2.3
	ω_4	133.2	0.1
	ω_5	141.7	0.4
	ω_6	183.8	5.5
	ω_7	263.7	0.4
	ω_8	428.3	0.6

	ω_9	470.6	2.9
	ω_{10}	548.6	8.9
	ω_{11}	885.9	268.3
	ω_{12}	1968.4	1468.8

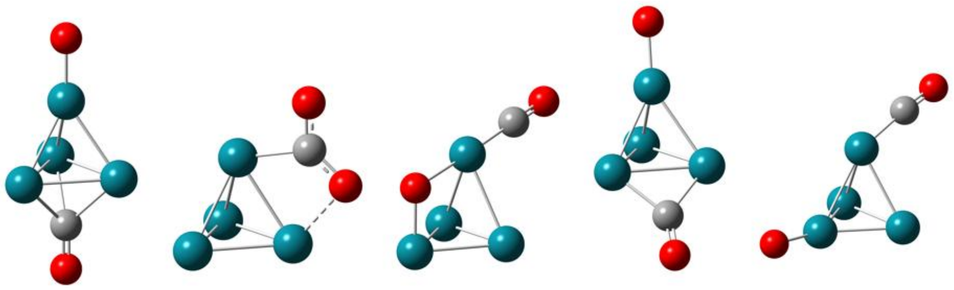

Table S2: Calculated harmonic frequencies of the $[Rh_4CO_2]^-$ structures displayed in Figure S3.

Structure	Mode	Frequency (cm^{-1})	Intensity (km/mol)
4A	ω_1	63.8	1.2
	ω_2	63.9	1.2
	ω_3	129.9	2.2
	ω_4	130.0	2.2
	ω_5	155.9	<0.1
	ω_6	156.0	<0.1
	ω_7	195.9	1.1
	ω_8	195.9	1.1
	ω_9	225.8	1.2
	ω_{10}	284.6	<0.1
	ω_{11}	419.0	0.1
	ω_{12}	419.2	0.1
	ω_{13}	437.4	0.4
	ω_{14}	848.5	319.4
	ω_{15}	1724.6	844.5
4B	ω_1	35.7	2.0
	ω_2	99.9	0.3
	ω_3	112.7	<0.1
	ω_4	136.5	1.5
	ω_5	164.2	1.8
	ω_6	169.0	<0.1
	ω_7	201.6	1.4
	ω_8	208.6	0.1
	ω_9	240.3	15.9
	ω_{10}	285.5	1.0
	ω_{11}	402.8	31.2
	ω_{12}	583.0	1.8
	ω_{13}	789.3	178.3
	ω_{14}	1231.6	558.6
	ω_{15}	1666.5	335.3
4C	ω_1	56.1	0.1
	ω_2	70.8	2.4
	ω_3	90.1	<0.1
	ω_4	94.4	5.9

	ω_5	136.0	2.7
	ω_6	162.5	0.1
	ω_7	165.7	2.2
	ω_8	184.4	1.1
	ω_9	277.9	0.6
	ω_{10}	429.6	25.9
	ω_{11}	526.5	1.1
	ω_{12}	532.4	2.8
	ω_{13}	554.6	16.0
	ω_{14}	675.0	115.6
	ω_{15}	1959.1	1698.1
4D	ω_1	33.2	3.1
	ω_2	76.8	1.7
	ω_3	99.9	2.0
	ω_4	126.5	0.4
	ω_5	126.7	1.7
	ω_6	153.0	1.0
	ω_7	187.5	0.4
	ω_8	206.0	0.1
	ω_9	210.7	0.3
	ω_{10}	259.1	0.9
	ω_{11}	403.4	4.8
	ω_{12}	474.2	0.8
	ω_{13}	492.9	0.9
	ω_{14}	837.1	332.2
	ω_{15}	1788.1	1030.3
4E	ω_1	52.4	1.4
	ω_2	60.0	4.8
	ω_3	88.3	0.3
	ω_4	117.9	0.5
	ω_5	137.1	0.5
	ω_6	143.6	0.6
	ω_7	178.9	0.1
	ω_8	182.6	<0.1
	ω_9	208.3	5.1
	ω_{10}	282.0	0.2
	ω_{11}	456.4	1.3
	ω_{12}	468.0	7.8
	ω_{13}	559.9	5.8
	ω_{14}	850.2	331.4
	ω_{15}	1967.3	1379.9

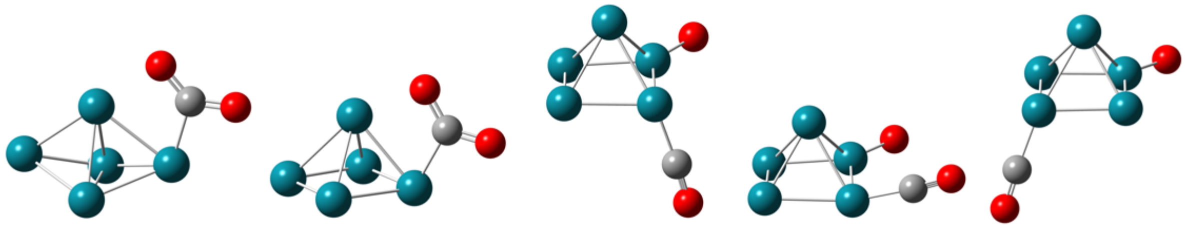

5A 5B 5C 5D 5E

Table S3: Calculated harmonic frequencies of the $[Rh_5CO_2]^-$ structures displayed in Figure S4.

Structure	Mode	Frequency (cm^{-1})	Intensity (km/mol)
5A	ω_1	39.7	3.3
	ω_2	76.1	1.0
	ω_3	99.7	0.2
	ω_4	110.8	1.6
	ω_5	126.1	1.7
	ω_6	149.3	0.2
	ω_7	158.3	0.9
	ω_8	171.6	0.1
	ω_9	183.9	0.6
	ω_{10}	211.1	1.2
	ω_{11}	229.1	5.0
	ω_{12}	238.8	7.4
	ω_{13}	274.4	1.0
	ω_{14}	406.0	38.3
	ω_{15}	590.4	2.3
	ω_{16}	791.7	211.5
	ω_{17}	1234.3	577.9
	ω_{18}	1677.7	422.2
5B	ω_1	40.1	3.5
	ω_2	76.3	0.5
	ω_3	94.4	0.3
	ω_4	112.2	0.7
	ω_5	116.9	1.9
	ω_6	120.5	1.7
	ω_7	158.3	<0.1
	ω_8	160.6	0.2
	ω_9	193.6	1.4
	ω_{10}	218.6	0.3
	ω_{11}	233.5	3.9
	ω_{12}	245.9	18.1
	ω_{13}	270.1	0.2
	ω_{14}	405.9	31.3
	ω_{15}	598.6	7.9
	ω_{16}	785.0	207.6
	ω_{17}	1222.6	477.2
	ω_{18}	1666.4	487.2
5C	ω_1	51.6	0.7
	ω_2	57.3	0.2

	ω_{3}	85.0	0.6
	ω_{4}	115.8	0.5
	ω_{5}	120.0	0.4
	ω_{6}	130.2	0.2
	ω_{7}	134.8	1.1
	ω_{8}	154.3	0.5
	ω_{9}	175.4	0.4
	ω_{10}	211.6	0.7
	ω_{11}	222.0	0.9
	ω_{12}	240.8	1.9
	ω_{13}	273.9	<0.1
	ω_{14}	451.0	1.5
	ω_{15}	461.3	3.1
	ω_{16}	529.1	8.3
	ω_{17}	861.8	387.3
	ω_{18}	1990.4	1405.7
5D	ω_{1}	46.1	0.1
	ω_{2}	62.9	0.3
	ω_{3}	70.0	1.1
	ω_{4}	86.8	4.6
	ω_{5}	107.4	0.8
	ω_{6}	129.3	1.3
	ω_{7}	139.2	0.6
	ω_{8}	157.5	0.8
	ω_{9}	171.3	0.7
	ω_{10}	199.3	1.2
	ω_{11}	232.2	3.9
	ω_{12}	250.4	1.8
	ω_{13}	270.6	0.3
	ω_{14}	456.7	6.4
	ω_{15}	464.2	2.4
	ω_{16}	530.1	10.4
	ω_{17}	851.1	371.7
	ω_{18}	1995.6	1504.8
5E	ω_{1}	51.8	1.3
	ω_{2}	54.4	1.0
	ω_{3}	87.4	1.1
	ω_{4}	128.6	1.0
	ω_{5}	132.1	0.1
	ω_{6}	135.6	0.3
	ω_{7}	143.6	0.7
	ω_{8}	159.3	0.7
	ω_{9}	165.8	<0.1
	ω_{10}	213.8	0.8
	ω_{11}	225.8	4.3
	ω_{12}	246.9	0.3
	ω_{13}	268.0	0.6
	ω_{14}	456.4	0.4
	ω_{15}	465.2	6.0
	ω_{16}	543.6	14.5

	ω_{17}	869.8	369.9
	ω_{18}	1970.6	1673.3

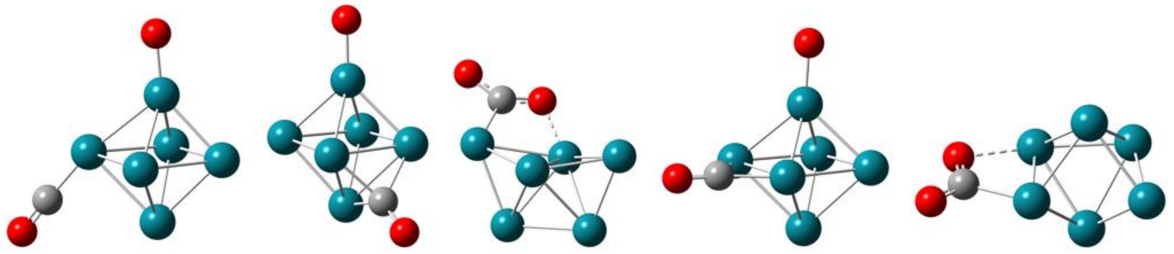

6A 6B 6C 6D 6E

Table S4: Calculated harmonic frequencies of the $[Rh_6CO_2]^-$ structures displayed in Figure S5.

Structure	Mode	Frequency (cm^{-1})	Intensity (km/mol)
6A	ω_1	39.3	1.2
	ω_2	55.5	2.3
	ω_3	100.6	<0.1
	ω_4	104.0	<0.1
	ω_5	114.9	<0.1
	ω_6	136.7	2.1
	ω_7	144.7	0.2
	ω_8	145.6	<0.1
	ω_9	147.1	2.2
	ω_{10}	158.8	1.0
	ω_{11}	195.5	0.1
	ω_{12}	205.3	<0.1
	ω_{13}	219.9	2.2
	ω_{14}	222.5	0.3
	ω_{15}	244.4	2.1
	ω_{16}	265.4	1.7
	ω_{17}	428.2	4.2
	ω_{18}	434.0	<0.1
	ω_{19}	522.9	19.8
	ω_{20}	874.8	431.6
	ω_{21}	1976.4	1576.6
6B	ω_1	90.5	3.5
	ω_2	94.6	2.5
	ω_3	105.0	0.7
	ω_4	117.2	1.1
	ω_5	129.2	0.2
	ω_6	132.3	0.3
	ω_7	136.9	1.0
	ω_8	153.9	1.2
	ω_9	178.6	0.8
	ω_{10}	185.2	0.3
	ω_{11}	194.0	0.5
	ω_{12}	205.1	1.8
	ω_{13}	217.3	1.1
	ω_{14}	222.2	0.6
	ω_{15}	238.3	0.8
	ω_{16}	255.8	0.4

	ω_{17}	396.8	0.9
	ω_{18}	415.3	4.5
	ω_{19}	436.8	4.4
	ω_{20}	865.9	370.6
	ω_{21}	1726.5	694.6
6C	ω_{1}	35.0	2.1
	ω_{2}	79.2	<0.1
	ω_{3}	85.0	0.4
	ω_{4}	93.8	0.2
	ω_{5}	109.9	1.2
	ω_{6}	142.4	2.7
	ω_{7}	150.5	0.7
	ω_{8}	156.3	0.4
	ω_{9}	167.7	0.5
	ω_{10}	175.2	0.8
	ω_{11}	184.0	0.1
	ω_{12}	206.6	0.3
	ω_{13}	217.6	2.8
	ω_{14}	244.6	14.8
	ω_{15}	257.3	0.2
	ω_{16}	279.0	1.9
	ω_{17}	406.3	24.6
	ω_{18}	592.0	2.2
	ω_{19}	787.0	242.4
	ω_{20}	1229.7	553.5
	ω_{21}	1701.9	358.6
6D	ω_{1}	37.3	0.6
	ω_{2}	91.9	0.2
	ω_{3}	95.4	0.3
	ω_{4}	109.3	<0.1
	ω_{5}	114.2	0.2
	ω_{6}	126.1	1.1
	ω_{7}	132.8	0.5
	ω_{8}	141.3	0.2
	ω_{9}	164.7	0.7
	ω_{10}	176.7	1.6
	ω_{11}	191.9	0.4
	ω_{12}	206.5	0.3
	ω_{13}	215.5	1.3
	ω_{14}	225.0	2.6
	ω_{15}	245.7	2.4
	ω_{16}	267.0	1.2
	ω_{17}	314.2	5.4
	ω_{18}	415.0	1.4
	ω_{19}	468.8	2.5
	ω_{20}	880.8	394.9
	ω_{21}	1842.3	994.2
6E	ω_{1}	41.5	3.6
	ω_{2}	45.9	0.4
	ω_{3}	105.7	0.1

	ω_4	106.5	0.5
	ω_5	123.1	0.2
	ω_6	128.5	<0.1
	ω_7	138.1	2.3
	ω_8	155.9	2.7
	ω_9	174.8	0.4
	ω_{10}	182.7	0.3
	ω_{11}	191.8	0.4
	ω_{12}	210.8	2.7
	ω_{13}	218.6	2.3
	ω_{14}	245.5	0.2
	ω_{15}	261.8	18.7
	ω_{16}	270.9	2.8
	ω_{17}	433.2	1.9
	ω_{18}	590.3	2.6
	ω_{19}	788.5	176.1
	ω_{20}	1187.4	376.8
	ω_{21}	1649.0	677.1

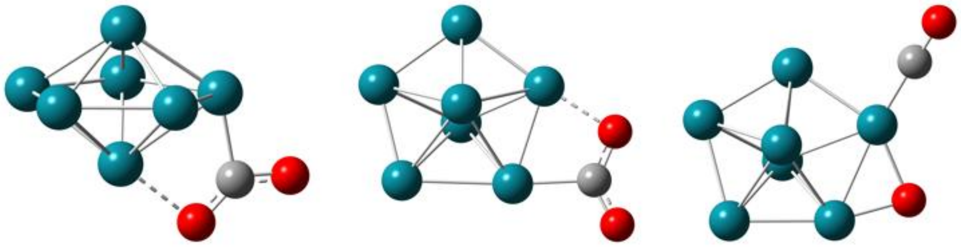

7A 7B 7C

Table S5: Calculated harmonic frequencies of the $[Rh_7CO_2]^-$ structures displayed in Figure 3.

Structure	Mode	Frequency (cm^{-1})	Intensity (km/mol)
7A	ω_1	42.3	1.5
	ω_2	88.1	0.7
	ω_3	92.1	<0.1
	ω_4	103.3	<0.1
	ω_5	115.5	<0.1
	ω_6	125.1	2.0
	ω_7	129.2	0.2
	ω_8	136.2	<0.1
	ω_9	140.9	<0.1
	ω_{10}	161.2	1.5
	ω_{11}	172.3	<0.1
	ω_{12}	174.9	2.0
	ω_{13}	191.1	<0.1
	ω_{14}	202.8	4.3
	ω_{15}	209.8	0.6
	ω_{16}	232.9	0.2
	ω_{17}	236.2	0.9
	ω_{18}	243.7	30.3
	ω_{19}	269.2	3.7
	ω_{20}	383.3	8.5
	ω_{21}	582.7	3.9
	ω_{22}	764.3	295.5
	ω_{23}	1221.6	503.7
	ω_{24}	1734.3	214.0
7B	ω_1	37.5	2.8
	ω_2	91.4	<0.1
	ω_3	99.1	0.1
	ω_4	101.4	0.9
	ω_5	124.1	0.2
	ω_6	130.3	<0.1
	ω_7	133.3	2.1
	ω_8	140.4	0.2
	ω_9	158.5	0.1
	ω_{10}	162.0	<0.1
	ω_{11}	172.4	1.1
	ω_{12}	178.7	2.3

	ω_{13}	189.1	0.1
	ω_{14}	198.9	4.2
	ω_{15}	211.4	1.3
	ω_{16}	225.2	10.0
	ω_{17}	256.0	3.5
	ω_{18}	263.4	2.5
	ω_{19}	272.1	5.7
	ω_{20}	441.6	17.7
	ω_{21}	567.6	4.3
	ω_{22}	786.5	217.3
	ω_{23}	1158.5	357.1
	ω_{24}	1679.2	714.4
7C	ω_{1}	11.9	0.4
	ω_{2}	37.0	1.4
	ω_{3}	58.5	0.4
	ω_{4}	75.8	0.4
	ω_{5}	97.7	0.2
	ω_{6}	112.5	3.9
	ω_{7}	121.2	0.2
	ω_{8}	127.2	0.9
	ω_{9}	142.8	0.2
	ω_{10}	147.9	3.5
	ω_{11}	154.7	0.2
	ω_{12}	177.5	2.5
	ω_{13}	186.3	1.4
	ω_{14}	194.7	2.7
	ω_{15}	207.6	0.4
	ω_{16}	224.7	0.5
	ω_{17}	227.3	1.2
	ω_{18}	254.6	0.8
	ω_{19}	347.5	7.6
	ω_{20}	425.1	16.3
	ω_{21}	460.3	2.5
	ω_{22}	533.4	12.8
	ω_{23}	625.7	147.2
	ω_{24}	1969.1	1930.9

5. Cartesian coordinates of calculated low-energy structures

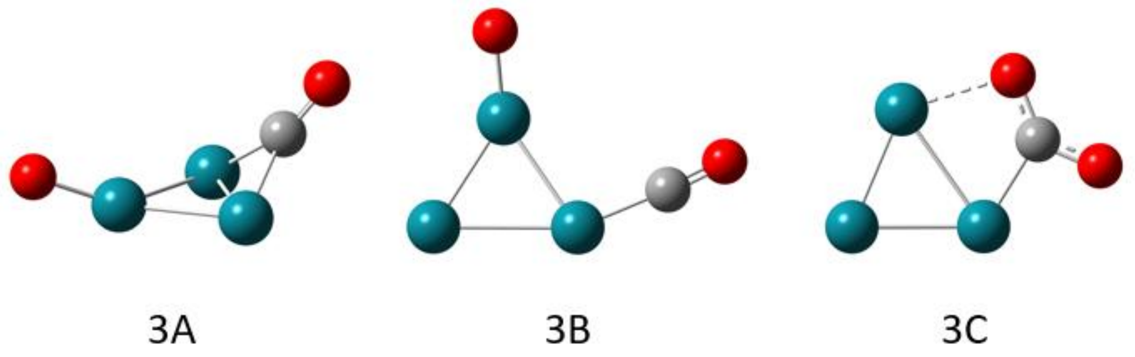

Table S6: Cartesian coordinates, in Å, of the $[Rh_3CO_2]^-$ structures displayed in Figure 3.

Structure	Atom	X	Y	Z
3A	Rh	0.597923	-1.24408	-0.26198
	Rh	-1.39292	0.000006	0.025935
	Rh	0.597953	1.244082	-0.26197
	C	1.731791	0.000007	0.648828
	O	2.652397	-4.7E-05	1.401776
	O	-2.84288	0.000016	0.912926
3B	Rh	-1.63602	-0.48701	0.099817
	Rh	-0.22458	1.365899	-0.091
	Rh	0.707057	-1.07436	-0.10501
	C	2.056544	0.219331	0.16293
	O	1.903482	1.462821	0.267386
	O	3.042786	-0.52784	0.15151
3C	Rh	-0.31977	1.143374	-0.12351
	Rh	-1.62102	-0.80595	-0.02329
	Rh	1.007753	-0.85206	0.106866
	C	2.661528	-0.18794	-0.05006
	O	3.702988	0.308499	-0.19794
	O	-0.4508	2.727303	0.460146

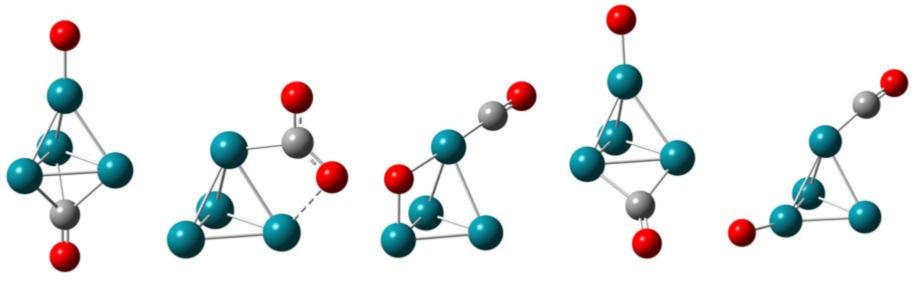

Table S7: Cartesian coordinates, in Å, of the $[Rh_4CO_2]^-$ structures displayed in Figure S3.

Structure	Atom	X	Y	Z
4A	Rh	0.438963	0.443917	-1.377072
	Rh	0.438889	-1.414540	0.304089
	Rh	-1.531490	-0.000008	-0.000037
	Rh	0.438947	0.970619	1.072978
	O	-3.249411	0.000128	0.000124
	C	1.862196	-0.000039	0.000018
	O	3.060400	-0.000030	0.000098
4B	Rh	-1.241316	-0.192003	-1.207423
	Rh	-1.235839	-0.198783	1.210855
	Rh	0.281806	1.398658	0.001605
	Rh	0.854313	-1.081193	-0.005817
	C	2.412724	0.002651	0.000292
	O	3.303755	-0.853918	0.001603
	O	2.430028	1.264361	0.002569
4C	Rh	1.801230	0.601731	0.000005
	Rh	0.169733	-0.931640	1.188968
	Rh	0.169610	-0.931036	-1.189277
	Rh	-1.186631	0.962458	0.000277
	C	-2.796445	0.167204	-0.000090
	O	0.567944	1.910854	0.000441
	O	-3.836535	-0.357272	-0.000216
4D	Rh	-0.788045	0.220856	-1.281022
	Rh	-0.788146	0.219682	1.281200
	Rh	1.368634	0.715342	0.000352
	Rh	0.571112	-1.561061	-0.000673
	O	2.952673	1.385691	0.000565
	C	-2.181960	0.420960	0.000137
	O	-3.361202	0.577731	0.000143
4E	Rh	0.038088	-1.293206	0.895231
	Rh	-1.050882	-0.000395	-1.006284
	Rh	1.534015	-0.000099	-0.408871
	Rh	0.037995	1.293862	0.894285
	O	2.790204	-0.000641	-1.578590

	C	-2.766913	-0.000239	-0.528946
	O	-3.860615	-0.000089	-0.130478

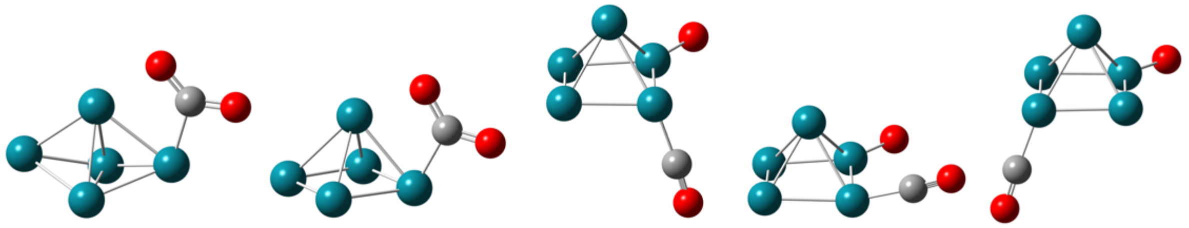

5A 5B 5C 5D 5E

Table S8: Cartesian coordinates, in Å, of the $[Rh_5CO_2]^-$ structures displayed in Figure S4.

Structure	Atom	X	Y	Z
5A	Rh	2.211941	0.488638	0.038090
	Rh	0.560803	-0.726796	1.301361
	Rh	0.512076	-0.808906	-1.303644
	Rh	-0.200998	1.324829	-0.097762
	Rh	-1.638251	-0.785473	0.025256
	C	-2.707601	0.786310	0.065888
	O	-2.256820	1.959594	0.022384
	O	-3.843809	0.306525	0.134637
5B	Rh	0.457685	-1.693285	-0.402547
	Rh	-1.287415	0.000753	-0.952308
	Rh	2.057076	0.000329	0.085488
	Rh	-0.251493	-0.001255	1.364480
	Rh	0.459808	1.692531	-0.401223
	C	-2.648726	0.001610	0.402012
	O	-2.373191	-0.000233	1.635010
	O	-3.715855	0.004240	-0.214654
5C	Rh	1.451293	-0.438668	0.536788
	Rh	-0.920497	0.069311	1.404038
	Rh	-0.475831	-1.782119	-0.300997
	Rh	0.286292	1.574625	-0.191352
	Rh	-1.469176	0.187253	-1.085199
	O	0.539528	3.262161	-0.175223
	C	2.846678	-0.568589	-0.604027
	O	3.670015	-0.644232	-1.415202
5D	Rh	1.074577	-1.033783	-0.545616
	Rh	-0.216263	-0.239017	1.532337
	Rh	-1.423344	-1.320162	-0.417358
	Rh	0.733391	1.391501	-0.245909
	Rh	-1.576773	1.035899	-0.307641
	O	1.842872	2.693615	-0.157331
	C	2.840938	-0.999776	-0.152075
	O	3.948741	-1.012500	0.182434
5E	Rh	0.019999	1.675798	0.218117
	Rh	0.564926	-0.001623	-1.541113
	Rh	-1.717382	-0.000648	-0.302136

	Rh	1.617479	0.001125	0.721834
	Rh	0.021022	-1.675652	0.221107
	O	3.207708	0.002724	1.338398
	C	-3.016181	0.000923	0.933534
	O	-3.792068	0.002208	1.798777

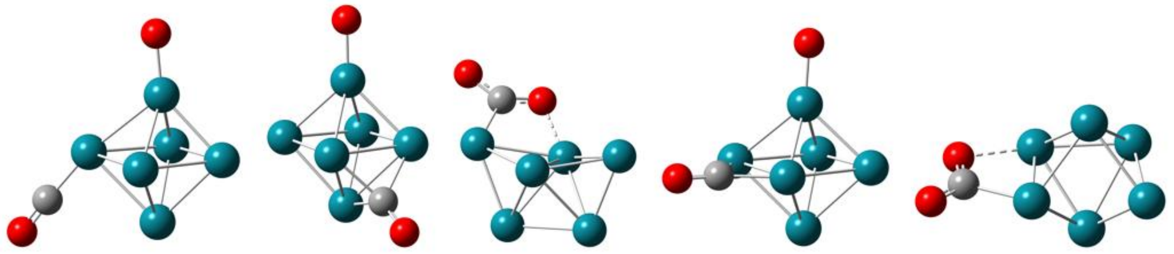

Table S9: Cartesian coordinates, in Å, of the $[Rh_6CO_2]^-$ structures displayed in Figure S5.

Structure	Atom	X	Y	Z
6A	Rh	-1.346823	1.104588	0.000254
	Rh	1.295110	1.189874	0.000750
	Rh	0.101635	-0.092124	-1.742879
	Rh	-0.950042	-1.536248	-0.001052
	Rh	1.574549	-1.210637	0.000006
	Rh	0.101165	-0.093906	1.742612
	O	2.199668	2.629666	0.000344
	C	-3.108736	0.716462	0.000820
	O	-4.230835	0.424285	0.000779
6B	Rh	-0.755322	-1.237894	1.077594
	Rh	1.461617	-0.936159	-0.192154
	Rh	0.828963	0.773224	1.491552
	Rh	-1.356848	1.174105	0.252553
	Rh	0.840711	1.433187	-0.991180
	Rh	-0.651070	-0.622974	-1.485510
	O	2.877998	-1.874099	-0.377307
	C	-2.175355	-0.633776	-0.159775
	O	-3.316769	-0.932688	-0.362674
6C	Rh	1.862031	-0.845368	-0.516367
	Rh	0.295187	-0.162266	1.408879
	Rh	-0.488014	-1.793575	-0.418291
	Rh	-0.168635	0.729261	-0.948028
	Rh	-2.120015	-0.139521	0.519258
	Rh	1.975971	1.402379	0.287575
	C	-2.626635	1.380815	-0.520500
	O	-1.899011	1.924702	-1.388432
	O	-3.761468	1.590816	-0.094471
6D	Rh	-0.996379	-0.020501	1.354799
	Rh	0.280257	1.597741	-0.015887
	Rh	-1.330912	0.011166	-1.188328
	Rh	0.075113	-1.857715	-0.000405
	Rh	1.279329	-0.192488	-1.395081
	Rh	1.626154	-0.153910	1.154797

	O	0.502563	3.280654	-0.060143
	C	-2.623492	0.081276	0.430327
	O	-3.786229	0.121741	0.244229
6E	Rh	-0.298614	-0.313154	1.639108
	Rh	0.868386	1.378017	0.170686
	Rh	-1.686822	1.199571	0.082645
	Rh	-1.635374	-1.340548	-0.102286
	Rh	-0.256622	0.084167	-1.690008
	Rh	1.373896	-1.071306	-0.121799
	C	3.015623	-0.068001	0.020563
	O	4.002565	-0.790995	-0.088012
	O	2.933441	1.197796	0.194392

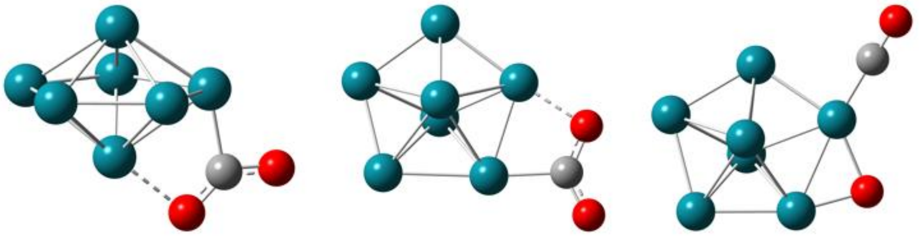

7A 7B 7C

Table S10: Cartesian coordinates, in Å, of the $[Rh_7CO_2]^-$ structures displayed in Figure 3.

Structure	Atom	X	Y	Z
7A	Rh	1.885218	-1.20671	-0.37341
	Rh	-0.3787	2.009846	0.292328
	Rh	0.575409	-0.00036	1.518713
	Rh	-0.3824	-2.00942	0.292063
	Rh	-1.9277	0.001069	0.656596
	Rh	-0.19553	0.000555	-1.35312
	Rh	1.888805	1.203859	-0.3733
	C	-2.78428	0.002189	-1.08242
	O	-3.95492	0.003582	-0.71606
	O	-2.1981	0.001268	-2.1839
7B	Rh	0.875746	2.082358	-0.0001
	Rh	-1.33201	-1.39741	-4.6E-05
	Rh	0.226336	0.008935	1.46927
	Rh	2.323762	0.167003	0.000163
	Rh	1.082189	-1.9681	-0.00016
	Rh	0.226433	0.009136	-1.46909
	Rh	-1.59683	1.084132	-5.6E-05
	C	-3.35632	0.214962	-7E-06
	O	-4.30122	0.987483	0.000542
	O	-3.3382	-1.07028	-0.00041
7C	Rh	1.634311	-1.61167	0.071897
	Rh	-2.00788	0.42268	-0.08989
	Rh	0.529453	0.006498	-1.53658
	Rh	2.181873	0.85218	0.249398
	Rh	-0.04942	1.997711	0.015577
	Rh	0.130014	-0.08549	1.455023
	Rh	-0.82014	-1.75348	-0.20633
	C	-3.53718	-0.47545	0.252572
	O	-4.51858	-1.03052	0.529079
	O	-1.81851	2.352194	-0.48847

6. Harmonic analysis and cartesian coordinates of key structures in the potential energy surface Figure 5

Table S11: Calculated harmonic frequencies of transition state 1 (TS1) on the Rh_3^- + CO_2 reactive potential energy surface (*i.e.,* Figure 5).

Structure	Mode	Frequency (cm^{-1})	Intensity (km/mol)
Singlet	ω_1	92.7i	4.3
	ω_2	66.3	0.5
	ω_3	103.5	1.0
	ω_4	167.2	0.4
	ω_5	219.7	1.6
	ω_6	303.8	3.0
	ω_7	325.3	6.8
	ω_8	367.3	31.3
	ω_9	580.5	1.3
	ω_{10}	766.5	230.1
	ω_{11}	1201.5	535.6
	ω_{12}	1768.4	319.3
Triplet	ω_1	71.2i	4.0
	ω_2	40.6	0.2
	ω_3	90.5	0.9
	ω_4	143.6	1.7
	ω_5	171.6	0.2
	ω_6	230.0	5.6
	ω_7	317.0	4.9
	ω_8	363.7	21.4
	ω_9	564.5	3.6
	ω_{10}	749.1	306.1
	ω_{11}	1195.5	549.1
	ω_{12}	1786.5	350.2
Quintet	ω_1	30.3i	2.0
	ω_2	54.2	0.9
	ω_3	87.9	0.2
	ω_4	128.8	2.2
	ω_5	177.6	2.0
	ω_6	264.0	1.1
	ω_7	310.7	6.6
	ω_8	366.7	15.6

	ω_9	573.8	1.7
	ω_{10}	744.1	343.9
	ω_{11}	1184.9	550.8
	ω_{12}	1809.6	486.3
Septet	ω_1	22.4i	3.4
	ω_2	58.0	2.6
	ω_3	64.8	0.9
	ω_4	94.8	1.5
	ω_5	172.5	0.1
	ω_6	258.8	0.5
	ω_7	301.4	2.7
	ω_8	350.7	6.8
	ω_9	567.7	20.9
	ω_{10}	718.5	507.7
	ω_{11}	1173.3	630.5
	ω_{12}	1872.0	581.4

Table S12: Calculated harmonic frequencies of transition state 2 (TS2) on the Rh_3^- + CO_2 reactive potential energy surface (*i.e.,* Figure 5).

Structure	Mode	Frequency (cm^{-1})	Intensity (km/mol)
Singlet	ω_1	382.1i	50.8
	ω_2	58.9	0.5
	ω_3	136.5	0.2
	ω_4	164.2	1.9
	ω_5	192.0	3.1
	ω_6	201.4	1.7
	ω_7	280.8	1.2
	ω_8	348.6	0.5
	ω_9	450.9	6.8
	ω_{10}	518.2	5.7
	ω_{11}	768.4	126.8
	ω_{12}	1951.7	1016.3
Triplet	ω_1	403.8i	40.6
	ω_2	54.0	1.1
	ω_3	122.4	1.9
	ω_4	135.2	2.7
	ω_5	173.0	<0.1
	ω_6	197.1	7.5
	ω_7	238.9	1.2
	ω_8	321.3	4.5
	ω_9	444.6	10.9
	ω_{10}	509.5	1.6
	ω_{11}	775.0	98.4
	ω_{12}	1911.1	997.8
Quintet	ω_1	446.1i	62.1
	ω_2	40.8	5.7
	ω_3	101.7	8.0
	ω_4	137.3	12.7
	ω_5	171.4	11.6
	ω_6	207.4	15.1
	ω_7	231.8	9.7
	ω_8	289.4	8.0
	ω_9	388.3	2.5
	ω_{10}	508.5	2.0
	ω_{11}	715.8	232.7
	ω_{12}	1803.3	1242.5
Septet	ω_1	501.9i	43.0
	ω_2	44.0	0.4
	ω_3	102.5	3.2
	ω_4	147.5	2.1
	ω_5	160.6	4.2
	ω_6	183.0	1.8
	ω_7	247.5	1.2

	ω_8	306.6	3.4
	ω_9	391.9	0.7
	ω_{10}	430.4	19.5
	ω_{11}	717.1	122.4
	ω_{12}	1933.0	956.9

Table S13: Cartesian coordinates, in Å, of transition state 1 (TS1) on the Rh_3^- + CO_2 reactive potential energy surface (*i.e.,* Figure 5).

Structure	Atom	X	Y	Z
Singlet	Rh	1.606302	-0.56965	0.254215
	Rh	0.430344	1.25705	-0.25649
	Rh	-0.81019	-0.85831	-0.21897
	C	-2.1992	0.246957	0.455893
	O	-2.27078	1.287899	1.082684
	O	-2.97863	-0.51177	-0.18012
Triplet	Rh	1.604690	-0.571661	0.249134
	Rh	0.330765	1.360441	-0.226675
	Rh	-0.710003	-0.887335	-0.239480
	C	-2.187570	0.150417	0.427139
	O	-2.374513	1.196982	1.010252
	O	-2.877981	-0.755419	-0.109864
Quintet	Rh	1.575747	-0.701531	0.258090
	Rh	0.509287	1.375788	-0.197459
	Rh	-0.796781	-0.781961	-0.272170
	C	-2.315538	0.201204	0.403248
	O	-2.567586	1.228081	0.986742
	O	-2.942184	-0.773151	-0.099273
Septet	Rh	1.726575	-0.709082	0.251611
	Rh	0.601744	1.273883	-0.163922
	Rh	-0.896551	-0.583802	-0.287007
	C	-2.625402	0.092640	0.385107
	O	-3.079755	0.996703	1.026264
	O	-3.004888	-0.959298	-0.193929

Table S14: Cartesian coordinates, in Å, of transition state 2 (TS2) on the Rh_3^- + CO_2 reactive potential energy surface (*i.e.,* Figure 5).

Structure	Atom	X	Y	Z
Singlet	Rh	1.498844	0.495376	0.42653
	Rh	0.158324	-1.29484	-0.30736
	Rh	-0.66432	0.996213	-0.53607
	C	-1.93071	0.312278	0.584924
	O	-1.29059	-1.71325	0.608899
	O	-2.84613	0.372298	1.297456
Triplet	Rh	1.669643	0.391999	0.380970
	Rh	0.021740	-1.299196	-0.318416
	Rh	-0.635221	1.054794	-0.457520
	C	-2.030171	0.351703	0.521591
	O	-1.392122	-1.547637	0.709748
	O	-3.026164	0.453630	1.120745
Quintet	Rh	-1.725622	0.128555	0.129284
	Rh	0.476638	1.160527	-0.289132
	Rh	0.189578	-1.344826	-0.059367
	C	1.815626	-0.381822	0.160807
	O	1.653601	1.298755	1.085486
	O	2.943836	-0.698828	0.026996
Septet	Rh	1.578032	0.439747	0.353058
	Rh	0.143191	-1.383277	-0.236572
	Rh	-0.608098	1.131559	-0.412222
	C	-2.101426	0.289215	0.434342
	O	-1.581039	-1.588385	0.317681
	O	-3.104221	0.313813	1.020079

Table S15: Relative energy (ΔE_0) as indicated in Figure 5 of Mol_{Min} compared to $Diss_{Min}$; negative values indicate thermodynamic preference for dissociation of the CO_2 moiety.

**Cluster**	**ΔE_0 / eV**
Rh3-	-0.16
Rh4-	-0.02
Rh5-	0.21
Rh6-	-0.12
Rh7-	0.27

7. Data analysis, power correction and cross-section determination

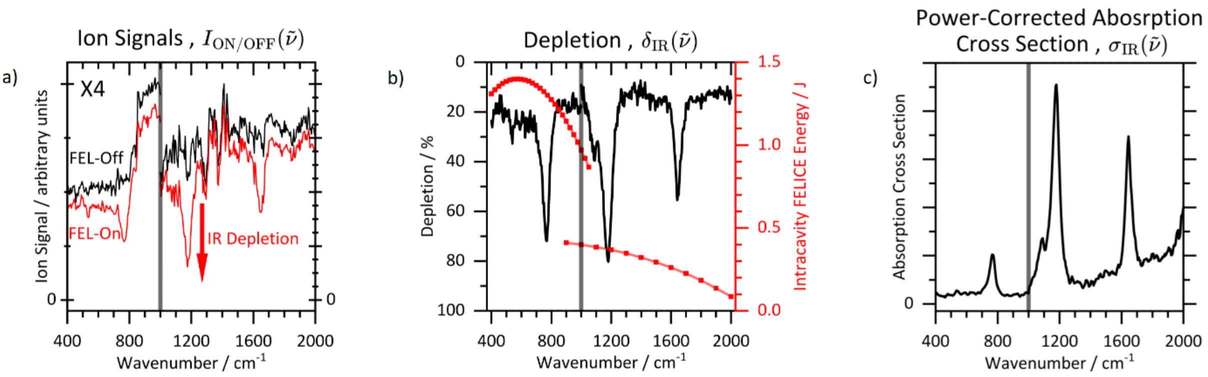

Figure S6: Key stages in producing IR-MPD spectra from raw mass spectrometric ion signals using the $[Rh_9CO_2]^-$ species as an example. Frequency-dependent IR depletions can be seen in FEL-on trace when overlayed on the FEL-off data, as shown in panel (a). The depletion spectrum can then be seen in panel (b), with the FELICE intracavity power curves in both regions also shown. The depletion spectrum is power-corrected to yield an IR-MPD absorption cross-section as shown in panel (c).
